# Accuracy of multiple sequence alignment methods in the reconstruction of transposable element families

**DOI:** 10.1093/nargab/lqac040

**Published:** 2022-05-17

**Authors:** Robert Hubley, Travis J Wheeler, Arian F A Smit

**Affiliations:** Institute for Systems Biology, Seattle, WA 98109, USA; Department of Computer Science, University of Montana, Missoula, MT 59801, USA; Institute for Systems Biology, Seattle, WA 98109, USA

## Abstract

The construction of a high-quality multiple sequence alignment (MSA) from copies of a transposable element (TE) is a critical step in the characterization of a new TE family. Most studies of MSA accuracy have been conducted on protein or RNA sequence families, where structural features and strong signals of selection may assist with alignment. Less attention has been given to the quality of sequence alignments involving neutrally evolving DNA sequences such as those resulting from TE replication. Transposable element sequences are challenging to align due to their wide divergence ranges, fragmentation, and predominantly-neutral mutation patterns. To gain insight into the effects of these properties on MSA accuracy, we developed a simulator of TE sequence evolution, and used it to generate a benchmark with which we evaluated the MSA predictions produced by several popular aligners, along with Refiner, a method we developed in the context of our RepeatModeler software. We find that MAFFT and Refiner generally outperform other aligners for low to medium divergence simulated sequences, while Refiner is uniquely effective when tasked with aligning high-divergent and fragmented instances of a family.

## INTRODUCTION

The ongoing explosion in the number of sequenced organisms highlights the need for reliable and thorough automated genome annotation pipelines. Most of the vertebrate genome finds its ultimate origin in transposable elements (TEs) (1–5), which have an enormous impact on genome activity and evolution (6–9). Due to the volume and diversity of TEs, complete annotation of genomes depends on accurate identification and modeling of TE families (10). A central aspect of that process is the gathering of instances of each family, and the creation of multiple sequence alignments (MSAs) of those instances; these MSAs are often used to derive a consensus sequence (1,11–13) and a profile hidden Markov model (pHMM) (14) for each family. Profile HMMs have been demonstrated to outperform consensus sequences for the identification of distant copies (15), however consensus sequences remain an important tool for interpreting sequence features such as open reading frames, splice sites, and transcription regulatory sites. In addition, many analysis tools are tailored to work with sequences rather than pHMMs and therefore are the focus of this work. Regardless of the sequence modeling methods, a high quality family-level TE MSA is critical for sensitive annotation of genomic copies, precise classification of TE families, reconstruction of encoded proteins, and family age estimation; this motivates an intense interest in the accuracy of methods producing MSAs for these sequence families.

Computational MSA approaches seek to optimize one of several scoring models, and an optimal solution of commonplace models is computationally intractable (16). Over the years, a multitude of MSA tools have been developed, each employing its own set of heuristics for achieving good alignment speed. The combination of heuristic and scoring functions leads to varying alignment accuracy. The alignment of multiple TE instances poses unique challenges, in that these copies can exhibit high sequence divergence, are often very fragmentary, and are dominated by neutral mutation patterns. Here, we seek to evaluate the efficacy of several commonly used tools in recovering accurate MSAs of neutrally evolving fragments of transposable element sequences.

TEs are prodigious generators of repetitive sequences in most genomes; their relationships can be difficult to recover due to rapid lineage bursts, complex recombination histories, and high rates of neutral mutation. The generation of an MSA from copies of a TE family is an important step in reconstructing the ancestral state of the TE and generating sequence models for genome annotation (15). TE sequences complicate alignment in several important ways: (i) Instances are often fragmented due to poor insertion fidelity, large deletions, or interruptions by insertions of other TEs. (ii) Due to their mostly neutral decay, there are generally no conserved regions that can anchor the alignment or open reading frames free from indel accumulation. (iii) Copies are often derived from a TE that was rapidly evolving in a genome; therefore, they represent a mixture of ancestral forms. (iv) Low complexity regions and internal repetition are common features. 5. The oldest detectable TE copies have accumulated over 35% substitutions since their arrival and given their neutral decay, have a substitution level of more than 70% between copies. In addition, rarer nonlinear events such as microduplication and inversion can further confound alignment.

Most MSA tools follow the *progressive alignment* approach, whereby a guide tree is estimated from the unaligned sequences and used to control the order in which sequences are merged into an increasingly complete MSA (17). These approaches often re-estimate the guide tree from the MSA and iterate this process until convergence. All tools evaluated except Refiner and Dialign employ this general framework. The differentiating factor for many of these tools is the objective function for scoring the pairwise alignments. Clustal Omega (18), Muscle (19), Kalign (20) and Dialign-TX (21) employ matrix-based scoring schemes to either pairs of sequence symbols or a column profile. T-Coffee (22) introduced a consistency-based scoring scheme in which a library of global/local pairwise alignments is used to generate position-specific scoring matrices (PSSMs) for the progressive alignment phase. Variations on this approach were later adopted by ProbCons (23), and MAFFT (24). Dialign bypasses the construction of a guide tree, and instead constructs an MSA by assembling pairwise collinear segment-to-segment alignments. Refiner (25), based on an ad-hoc approach that we have employed in the curation of TE families for over a decade, follows a pattern that we call iterative transitive alignment: all sequences are locally aligned to a single template sequence and the MSA is produced by aligning sequences to each other based on their alignments to the common template. The sequence representing the centroid of the set is used as the initial template. When complete, a consensus is computed from the resulting MSA, and the process is iterated using this new consensus as the template; iteration continues until convergence.

Evaluation of MSA tool accuracy usually depends on protein benchmarks, consisting of real sequences (26–29), based on structural (PREFAB (19)) or hand-curated alignments(e.g. BAliBASE (30), SABmark (31), HomFam (32), HOMSTRAD (33) and OXBENCH (34)). BRaliBase (35)) stands out as a rare benchmark for nucleotide (RNA) alignment. Simulated sequence evolution datasets have also been used to evaluate MSA tools (36–39), providing the means to produce larger test sets, a wide range of sequence divergence characteristics, and supporting the generation of DNA-specific benchmarks. However, these previous studies focused on constrained sequences (36), protein simulations (37,38), or ignored the problem of working with fragmented sequences (36–39).

Sequence simulation tools have themselves evolved over time. Early efforts focused primarily on the generation of sequences along a fixed phylogeny, allowing for mutations based on time reversible substitution models (40–43). These led to more sophisticated evolvers with support for insertion and deletion (indels) mutations, empirical substitution matrices, and branch dependent mutation rates (44–50). Context dependent mutation rates have also been developed in some simulators; for instance the Evolver package (51) models special mutation rates for highly mutagenic CpG dinucleotides, and Trevolver (52) implements a triplet substitution model that accounts for first-order flanking effects.

**Figure 1.**
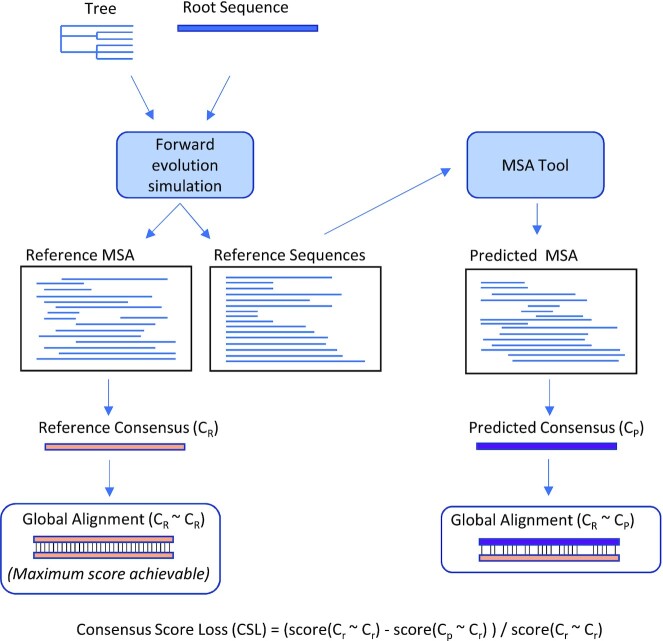
Consensus score loss metric—assessing the quality of a predicted MSA by comparing the consensus produced by the predicted MSA to the consensus of the true (reference) MSA through global alignment.

Several metrics have been widely used in the assessment of predicted MSA datasets. These include the Sum-of-Pairs Score (SPS, aka developer score) (53,54), Column Score (CS) (53), and the Alignment Metric Accuracy (AMA) metric (55). SPS is the fraction of aligned residue pairs in the reference alignment that are correctly aligned in the predicted alignment. The CS score is the fraction of aligned columns in the reference alignment that are perfectly reconstructed in the predicted alignment and is often used as a measure of specificity in reconstruction of the MSA. A single misaligned sequence in a MSA can decimate the CS score, so that CS provides limited power to discriminate between alignments of highly diverged sequences. The AMA metric is the fraction of characters in the reference alignment that are correctly arranged in the predicted alignment, either aligned to another character or not aligned (i.e. aligned to a gap character). This is an appealing metric, in that it penalizes overalignment in MSA (failure to allow gaps as appropriate), but it does not factor in gap position and can therefore be thrown off by radically different gap positions within the predicted MSA (56). Motivated by the standard practice of generating centroid sequence models (consensus sequences) for MSAs of TE families, herein we introduce a new metric, ‘consensus score loss’ (CSL). CSL assesses the quality of the MSA by comparing the consensus produced from a predicted MSA to the consensus derived from the reference MSA (Figure 1). We also demonstrate an additional approach for assessing the quality of an MSA in the case of TEs with coding capacity and apply it to natural copies of several ancient families of DNA transposons.

To the best of our knowledge, no formal evaluation of MSA tools has been previously conducted in the context of TE sequence families. To study this, we developed a new sequence simulator (TEForwardEvolve), and used it to generate a benchmark of simulated MSAs. Using this benchmark, supplemented with a small collection of ancient mammalian DNA Transposon families, we evaluated: MUSCLE, MAFFT, Dialign-TX, Kalign, FSA, Clustal Omega, ProbCons and T-Coffee, along with our Refiner approach. We demonstrate that MAFFT generally outperforms other generic alignment tools, and that our Refiner method produces comparable results for low-divergence sequences, and superior alignments when confronted with high levels of sequence fragmentation and sequence divergence.

## MATERIALS AND METHODS

### Tree generation

**Figure 2.**
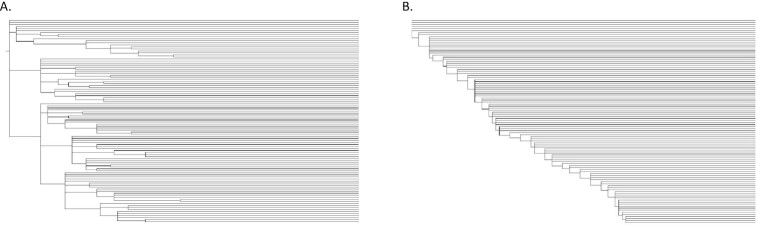
Phylogenetic trees for simulations: (**A**) random template phylogeny generated typical of DNA transposons, and (**B**) master gene phylogeny for LINE families.

A custom-made tool (genRandomTETrees.pl) was used to simulate phylogenetic trees for DNA Transposon and LINE TE families. For DNA Transposons, which exhibit a star-like phylogeny (Figure 2A), the tree is expanded by randomly choosing a parent node from the existing tree and appending a new child node with a random branch length between 0 and 10. Once the target number of extant nodes has been reached (100), the post-extinction phase of the sequence lifecycle is simulated by adjusting extant (leaf) node branch lengths to reach the target root-to-leaf length (100). Branch lengths in the tree do not equate to a specific unit of time; rather, they establish the relative duration of each branch. For the purposes of sequence simulation, the notional duration of a branch (i.e. the amount of mutation) is controlled by the simulator parameter ‘generations per unit time’ (GPUT).

To simulate master-gene model phylogenies such as seen in LINE families (Figure 2B), the tree is expanded by adding a randomly determined number of children (2–5) with randomly chosen branch lengths (0–5) to the current parent node (initially the root of the tree). One of the new children is randomly picked to be the new parent node (or ‘master gene’) and the process is iterated until the target number of extant nodes is reached (100). The branch lengths for extant nodes are then adjusted in a similar manner to the DNA transposon trees.

### Sequence simulation

While there are many sequence evolution simulators currently available, none provide all the features necessary for realistic TE sequence simulation in one package: nucleotide simulation, indel simulation, context dependent (trinucleotide) substitution matrices, and fragmentation simulation. Inspired by the release of TRevolver (52), which supports tri-nucleotide substitution context, we developed TEForwardEvolve, which supplements tri-nucleotide substitution with simulation of indels and fragmentation.

For each class of TE, the simulator is provided a prototypical TE sequence (in this study: Tigger1/Charlie1 for DNA transposons and L2/CR1 for LINEs), a simulated phylogenetic tree, a context-dependent substitution rate matrix, indel parameters, the number of generations represented in the tree, and fragmentation parameters. The substitution rate matrix consists of all triplet pairs where the center base is allowed to change and the edge bases provide 1bp of flanking context (64 × 64 matrix); rates were derived from a study of 160 000 non-coding sites in a set of mammalian genomes (57). Indel lengths were modeled using a power law (Zipfian) probability distribution (insertion/deletion mean length = 1.7, insertion/deletion max length = 20) with an occurrence rate of 0.20 (insertion rate = 0.08, deletion rate = 0.12) relative to an average substitution rate of 1. Finally, fragmentation is simulated by selecting fragment sizes from a log normal distribution, a minimum fragment size and a randomly chosen start position to select only a portion of the parent sequence for duplication. Optionally, a minimum number of full-length sequences may be set such that fragmentation begins only after the minimum number of full-length sequences has been generated.

To study the impact of sequence substitution level, TEFowardEvolve was run with increasing values for the generations-per-unit-time (GPUT) simulation parameter (100–6000). This translates to an average Kimura divergence range of 1–52% for Tigger1 and 1–44% for L2 simulations. To study the impact of fragmentation, TEForwardEvolve was run with increasing levels of fragmentation (mean copy lengths ranging from 1200 down to 75, with a standard deviation of 300, minimum fragment size = 50, minimum full-length sequences = 2) at two substitution levels (GPUT 100, 3000). For each parameterization, 10 replicate simulations were run.

### MSA evaluation

For each sequence evolution simulation, TEForwardEvolve provides a reference MSA for comparison to the MSAs predicted by the alignment tools. The qscore (19) tool was used to compute various metrics on the predicted MSAs including SPS, which is the fraction of aligned residue pairs in the reference alignment that are correctly aligned in the predicted alignment.

We also define a new score metric ‘consensus score loss’ (CSL), which assesses the quality of the predicted MSA by comparing (aligning) the consensus derived from it to the consensus derived from the reference MSA. If the predicted MSA is accurate, its induced consensus will be highly similar to the consensus from the reference MSA, and an alignment of the two consensus sequences will produce a high score, whereas an inaccurate predicted MSA will produce a low score; CSL characterizes MSA quality by computing the amount of the optimal consensus alignment score that is lost by using a predicted MSA. Specifically: let C_p_ be the consensus sequence derived from the predicted MSA, and C_r_ the consensus sequence produced from the reference MSA (Figure 1). To measure similarity of C_p_ to C_r_, CSL aligns them to each other via Needleman-Wunch global pairwise alignment (58) C_p_ ∼ C_r_, and aligns C_r_ to itself to produce alignment C_r_ ∼ C_r_. If the predicted MSA is perfectly accurate, C_p_ will be identical to C_r_, so that score(C_p_ ∼ C_r_) = score(C_r_ ∼ C_r_). An inaccurate predicted MSA will cause score(C_p_ ∼ C_r_) < score(C_r_ ∼ C_r_). CSL quantifies this by calculating the fraction of the ideal score that is lost with the predicted alignment: (score(C_r_ ∼ C_r_) - score(C_p_ ∼ C_r_)) / score(C_r_ ∼ C_r_) In the case of an extremely poor predicted MSA, the predicted consensus may lead to a negative global alignment score(C_p_ ∼ C_r_), so that >100% of the score is lost. For the analysis presented here, alignment was performed with a custom scoring matrix and gap parameterization described in the supplemental materials.

### MSA tools and parameters

The MSA tools covered in this evaluation are shown in Table 1, including versions and any non-default parameter settings. No attempt was made to optimize parameters aside from ensuring that DNA specific defaults were used. For Kalign, the DNA/RNA default parameters were based on instructions on the software website (https://msa.sbc.su.se/cgi-bin/msa.cgi). For MAFFT, the linsi algorithm was chosen based on the guidance on the software website (https://mafft.cbrc.jp/alignment/software/).

**Table 1.** MSA Software evaluated—the version of each tool evaluated and any specific parameters provided

Tool	Version	Parameters
MUSCLE	v3.8.31	
MAFFT L-INS-i	v7.407	–localpair –maxiterate 1000
Dialign-TX	v1.02	
Kalign	v2.0.4	-gpo 80 -gpe 3 -tgpe 3 -bonus 0
FSA	v1.15.9	
Clustal Omega	v.1.2.4	
Refiner	v2.0.2a	
T-Coffee	v13.45.0.4846264	
Probcons	v1.12	

### Refiner methodology

Our Refiner method works by establishing a single template sequence, aligning all sequences to that template, then producing an MSA based on the way that those sequences align to the template (in what we call a transitive alignment). In the first pass, the template is chosen from the input sequences by picking the sequence with the best cumulative pairwise alignment score to all other sequences or roughly the centroid of the set. The resulting MSA is used to produce a consensus, which is used as the template for iterative rounds of transitive alignment, in the form of Expectation Maximization. The tool currently supports either RMBlast (59) or ABBlast (60) for pairwise alignment, although any sensitive aligner would suffice. This process is repeated until convergence; see (25,61) for details. The final reference sequence is the consensus for the family.

In this local-alignment strategy, characters that do not align to the template sequence are either arbitrarily aligned to each other (internal insertions) or not included in the alignment at all. This is not a problem in the context of RepeatModeler, since these are not part of high occupancy columns, so not part of the final consensus. For the purpose of MSA SPS evaluation, all characters must be present in the final alignment, so we simply add all such characters to the MSA such that they are not aligned to any other character.

The consensus caller used by Refiner employs two stages. The caller initially identifies the highest scoring character (‘A’,‘C’,‘G’,‘T’,‘N’ or ‘–’) for each column from the subset of sequences aligning over it. The first step uses a matrix that reflects observed neutral DNA substitution patterns and is similar to matrices developed for RepeatMasker. For organisms with CpG methylation, which causes high conversion of CG to CA and TG, a second pass evaluates all dinucleotides in the initial consensus sequence for reassignment to ‘CG’ by registering the frequency of the most common products of CpG mutation, aligned CA and TG dinucleotides (61).

## RESULTS

### Simulated trees and sequences

We chose two TE classes to simulate, the Long Interspersed Nuclear Element (LINE), and the DNA transposon, to determine if their starkly different phylogenies lead to differences in the relative performance of MSA tools. The relationship of DNA transposon copies (Figure 2A) is typically a (near) star phylogeny (62,63), with any branching occurring randomly and early on. This reflects the fact that, due to random selection of the genomic template by the transposase, class II transposons tend to exhibit a short burst of activity before going extinct (1,64), and leave many neutrally decaying copies in the genome.

DNA transposon star phylogenies may be contrasted with those of LINEs (Figure 2B), in which most copies are derived from a single dominant lineage of LINE TEs (the so-called master-gene model of evolution (65,66)); the resulting phylogenies approach those of pseudogenes of a rapidly evolving cellular gene (67). Phylogenetic trees approximating the evolutionary patterns of DNA transposons and LINE families were randomly generated using a custom-made tool (see Methods).

Sequences were simulated along these trees using a forward evolution sequence simulator seeded with a class-specific TE consensus sequence (see Materials and Methods). The DNA transposon sequence simulation was seeded with the Tigger1 family consensus (68), and the LINE tree was seeded with the L2 consensus (1). Simulation was run with ten replicates at 18 evolutionary time increments, producing 180 simulated sequence sets and reference MSAs (100 sequences each). The evolutionary time increments generated sequences ranging from 0.01 to 0.5 average Kimura (69) sequence divergence. Evaluation with Charlie1 (DNA transposon) and CR1 (LINE) produced similar results, and are presented in the supplementary material (S2.2; Figures 1 and 2).

### Alignment reconstruction accuracy

**Figure 3.**
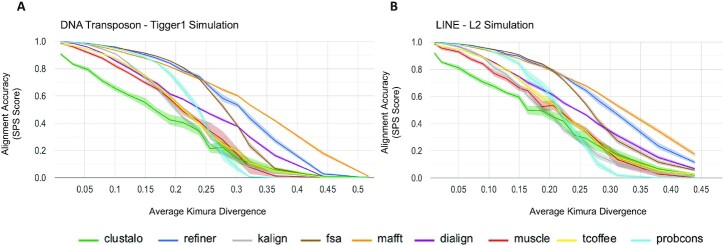
MSA accuracy with respect to sequence divergence. The MSA alignment accuracy for each method assessed using the sum-of-pairs (SPS) score over a wide range of sequence divergence. For each tool/divergence combination, 10 replicates were performed, with 100 sequences per replicate. The center line of each band shows the mean SPS score for the tool, while the surrounding shaded region shows the 95% confidence interval. (**A**) The Tigger1 DNA transposon family average SPS scores over 10 replicates. (**B**) The L2 LINE family average SPS scores over 10 replicates.

We computed SPS for all methods over a wide range of sequence divergence and for both TE classes (Figure 3). SPS results were significantly separated for both the DNA transposon simulations (*P*-value = 2.49e–22) and the LINE simulations (*P*-value = 1.98e–29; both p-values computed using the Kruskal–Wallis *H* test (70)). Furthermore, MAFFT and Refiner significantly outperformed other methods, according to a Wilcoxon signed rank test (71) see Supplementary material S1.1 for the full pairwise comparison table).

### Alignment as a basis for consensus sequences

**Figure 4.**
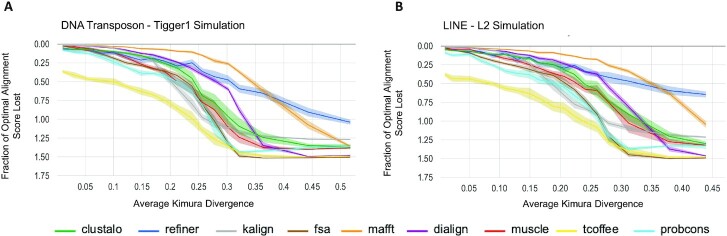
Accuracy of derived consensus sequence with respect to sequence divergence. Comparison of the predicted MSA-based consensus with the reference MSA-based consensus, for a range of sequence divergences. (**A**) For simulated Tigger1 sequences with variable levels of Kimura divergence, this plot shows the fraction (score(C_r_ ∼ C_r_)-score(C_p_ ∼ C_r_))/ score(C_r_ ∼ C_r_), which corresponds to how effective the computed MSA is at producing a consensus sequence (C_p_) that agrees with the one for the simulated sequence (C_r_). Scores are for the Needleman-Wunsch (NW) global alignment algorithm (see Materials and Methods). Center line of each band shows the mean loss of score for each tool, while the surrounding shaded region shows the 95% confidence interval. (**B**) The same fraction-of-optimal-score is captured, but for sequences simulated from L2.

Consensus sequences can be derived from MSAs by choosing the most likely ancestral base at each position in the MSA (considering only positions with high occupancy) (72); this has, for example, long been the source of family consensus sequences used in annotating TEs. When TE copies form a star phylogeny, which appears to be the case for most class II transposon copies in mammals (73), the consensus will be identical to the ancestral sequence of the active TE. In case of the LINE MSA, a consensus may approach an average of the evolving active TE. We computed the CSL measure for each tool at a variety of divergence levels (Figure 4), showing the extent to which computed alignments support recovery of accurate consensus sequences.

### Effect of sequence fragmentation

**Figure 5.**
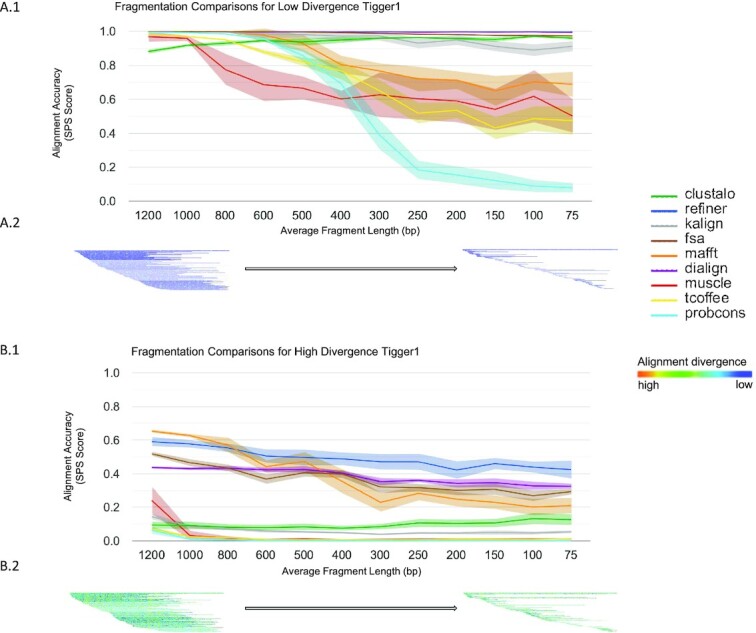
MSA accuracy with respect to sequence fragmentation. (**A.1**) The SPS results for simulations of Tigger1 (1% Kimura divergence) over increasing levels of sequence fragmentation. Fragment lengths are sampled from a distribution around the given mean (x-axis) with a standard deviation of 300. Center line of each band shows the mean SPS for each tool, while the surrounding shaded region shows the 95% confidence interval. (**A.2**) A visualization of the fragmentation of the reference MSA for the least fragmented and most fragmented datasets. Each line represents a single fragment; warmer colors represent higher sequence divergence over 10 bp windows in the alignment. (**B.1**) The SPS results for simulations of Tigger1 (28% Kimura divergence) over increasing levels of sequence fragmentation, with fragment length sampled as above. (**B.2**) Heatmap visualization of the fragmented MSA, as with (A.2), but for the higher divergence Tigger1 benchmark.

**Figure 6.**
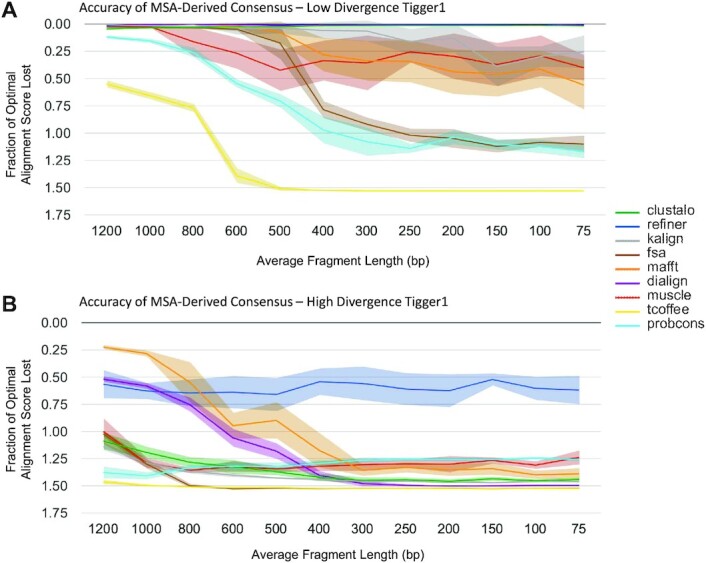
Accuracy of derived consensus with respect to sequence fragmentation. Comparison of the predicted MSA-based consensus with the reference MSA-based consensus, for fragmented sequences. (**A**) Fraction of ideal sequence alignment score, as in Figure 4; input sequences are low-divergence fragments (1% Kimura divergence) as from Figure 5A. (**B**) Same as in A, but with high-divergence fragment inputs (28% Kimura divergence) as from Figure 5B.

We evaluated the effect of fragmentation on MSA reconstruction as above, comparing the predicted MSA to the reference MSA, assessing both low divergence (1% avg Kimura divergence (69)) and high divergence (28% avg Kimura) sequences. The mean fragmentation size was varied from 75 to 1200 bp, based on observed fragmentation patterns in mammalian TE copies (Supplementary material S2.5). Figure 5 shows the effects of fragmentation level on SPS for a DNA transposon simulation and provides a visualization of the patterns for the fragmentation extremes. Most tools performed well on low-divergence sequences over a wide range of fragment sizes; at higher sequence divergence and fragmentation, Refiner outperforms all methods tested (Wilcoxon *P*-value ≤ 1.1e–11), with MAFFT, Dialign and FSA outperforming the rest. We also explored the effect of fragmentation on the accuracy of MSA-derived consensus sequences, as in the previous section (Figure 6). At high sequence divergence levels, Refiner was the only tool with partial retention of correct alignment score, showing that it effectively produces MSAs that yield accurate consensus sequences. We found similar results when we seeded the simulation with the LINE tree and the L2 sequence (Supplementary material S2.3).

### Comparison with natural sequence

**Figure 7.**
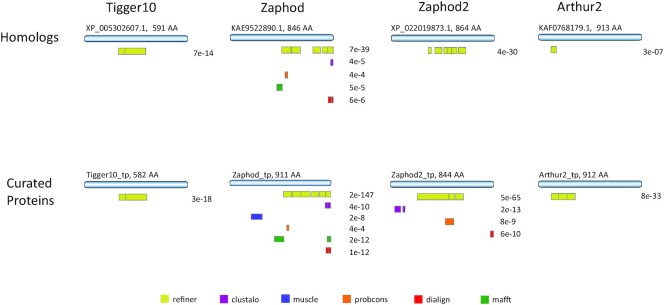
Protein reconstruction assessment for four mammalian TE families using 100 human-derived copies. A consensus model was built from each MSA and compared to the known curated transposase protein sequence and to distant homologs found in the NCBI NR protein database using blastx (matrix: BLOSUM62). For each method, the union of blastx results with *e* value <0.001 are plotted below the full-length database protein; the range of blastx coverage is captured in colored boxes, and the lowest *e*-value for the set is displayed.

We selected four DNA transposons: Zaphod (74), Zaphod2 (75), Tigger10 (76) and Arthur2 (77) to compare reconstruction accuracy at the protein level. These were selected for their extreme age (Tigger10 and Arthur2 predate the common ancestor of marsupials and placental mammals) and the high divergence of human copies to each other are expected to pose a challenge for MSA tools. For these families, there exist high quality manually created consensus sequences, supplemented by copies from reconstructed ancestral mammalian genomes (78). As DNA transposons, they are expected to have a star phylogeny, so that an accurate MSA should recreate the ORFs of the active elements. For each family, 100 annotated genomic instances from the human genome were sampled randomly from all members of the family, and aligned with each tool. A consensus was generated from each predicted MSA, then blastx (default: matrix = blosum62, E = 10.0, gap_open = 11, gap_ext = 1) was used to compare the consensus to the curated transposase proteins from our RepeatMasker Repeat Protein Database. To avoid the potential for circularity due to the fact that we were involved in both the curation of the protein sequence and the present assessment of alignment, we also searched the MSA consensi against homologs for these proteins found in the RefSeq NR database. For each comparison, the union of blastx results with an *e*-value <0.001 were plotted (Figure 7) against the full length protein. Though none of the MSA-derived consensus sequences is able to produce a full-length blastx match to the related protein sequence, one tool (Refiner) demonstrates clearly superior MSA-based blastx results: (i) only consensus sequences derived from the Refiner MSAs had matches for all elements and, (ii) Refiner-based blastx alignments were much longer and higher-scoring. This surprising result underscores the value of evaluating reverse translation quality among other metrics for TEs with coding regions.

## DISCUSSION

Using our new method TEForwardEvolve, we simulated the evolution of TE families over a wide variety of sequence divergence and fragmentation to investigate the impact on MSA prediction quality. The evaluated tools exhibited similar patterns of performance degradation for both TE classes as the sequence divergence was increased, with statistically significant performance differences among them. For full-length sequence inputs, MAFFT and Refiner maintained the highest alignment accuracy over the range of divergences. Surprisingly, the phylogenetic structure of the simulated trees did not produce a noticeable effect on alignment performance, suggesting that the results would hold for other TE classes.

SPS (aka the developer score) is an established and generally informative measure of global MSA reconstruction; however, it doesn’t appear to fully reflect the performance of MSA tools in the context of consensus sequence prediction. This can be seen most clearly in the performance of Refiner at high sequence divergences, where it is outcompeted by MAFFT’s SPS score while simultaneously producing superior accuracy of the MSA-derived consensus sequence. Local alignment appears to be key to explaining the difference. The consensus is negatively influenced by the tendency of many MSA tools to force mismatched sequence regions together in the MSA (55,79); local alignment avoids this at the expense of a complete alignment. In addition, short regions of misalignment in an MSA, while not penalized heavily by the SPS metric, can lead to incorrect consensus generation. For example: with highly fragmented sequences, FSA occasionally produced alignments where a majority of the sequences were incorrectly anchored at the start of the alignment for a short stretch (3 bp) followed by large gaps before continuing in roughly the correct location in the overall MSA (see Supplementary material S2.4). In cases such as this, the alignment artifact would cause a consensus caller to consider the sequences contained in the gap as probable insertions and generate a dramatically shortened consensus.

Our results suggest that even low levels of fragmentation, when combined with higher sequence divergence, poses a significant challenge to MSA tools. Faced with fragmentary input, Refiner produced MSAs that were significantly more accurate than other tools, highlighting the value of its iterative transitive alignment approach for this challenging form of input. For this study, fragmentation is simulated as a random process, whereas fragmentation processes exhibit biases for many TE classes (e.g. 5’ truncation in LINE elements, or the generation of solo LTRs through recombination). We plan to extend our simulator, TEFowardEvolve, to explore the effect of these fragmentation architectures on MSA prediction as well as additionally consider SINE and LTR TE classes.

Finally, simulation results were validated with an evaluation using manually curated protein sequences, known homologs, and instances of the TE family sequences that once encoded them. Only one tool (Refiner) was consistently successful in computing an MSA-based consensus that matched the corresponding proteins in a blastx search. The classification of a TE family is greatly facilitated by comparing to existing TE protein databases, underscoring the importance of an accurate MSA reconstruction. This analysis was restricted to DNA transposons due to the limited availability of ancient TE protein reconstructions. Natural sequences present the most compelling benchmark; however, it is difficult to obtain large enough samples for a complete parametric analysis. We plan to further extend this aspect of the benchmark as a complement to the simulated datasets.

## DATA AVAILABILITY

TEForwardEvolve, genRandomTETrees, analysis scripts, Nextflow (80) job management scripts, along with simulated data generated as part of this study, are available on github at: https://github.com/Dfam-consortium/TEForwardEvolve, release tag ‘1.0-paper’. MSA tool results, graphs and alignment visualizations may be downloaded from: https://www.dfam.org/web_download/Publications/MSABench2021.

## Supplementary Material

lqac040_Supplemental_Files

## References

[1] Smit A.F. The origin of interspersed repeats in the human genome. Curr. Opin. Genet. Dev. 1996; 6:743–748. 8994846 10.1016/s0959-437x(96)80030-x

[2] Smit A.F.A. Interspersed repeats and other mementos of transposable elements in mammalian genomes. Curr. Opin. Genet. Dev. 1999; 9:657–663. 10607616 10.1016/s0959-437x(99)00031-3

[3] Lander E.S. , Linton L.M., Birren B., Nusbaum C., Zody M.C., Baldwin J., Devon K., Dewar K., Doyle M., FitzHugh W. et al. Initial sequencing and analysis of the human genome. Nature. 2001; 409:860–921. 11237011 10.1038/35057062

[4] Kazazian H.H. Mobile elements: drivers of genome evolution. Science. 2004; 303:1626–1632. 15016989 10.1126/science.1089670

[5] Jurka J. , Kapitonov V.V., Kohany O., Jurka M.V. Repetitive sequences in complex genomes: structure and evolution. Annu. Rev. Genomics Hum. Genet. 2007; 8:241–259. 17506661 10.1146/annurev.genom.8.080706.092416

[6] Rebollo R. , Horard B., Hubert B., Vieira C. Jumping genes and epigenetics: towards new species. Gene. 2010; 454:1–7. 20102733 10.1016/j.gene.2010.01.003

[7] Jacobs F.M.J. , Greenberg D., Nguyen N., Haeussler M., Ewing A.D., Katzman S., Paten B., Salama S.R., Haussler D. An evolutionary arms race between KRAB zinc-finger genes ZNF91/93 and SVA/L1 retrotransposons. Nature. 2014; 516:242–245. 25274305 10.1038/nature13760 PMC4268317

[8] Farré M. , Narayan J., Slavov G.T., Damas J., Auvil L., Li C., Jarvis E.D., Burt D.W., Griffin D.K., Larkin D.M. Novel insights into chromosome evolution in birds, archosaurs, and reptiles. Genome Biol. Evol. 2016; 8:2442–2451. 27401172 10.1093/gbe/evw166 PMC5010900

[9] Wylie A. , Jones A.E., D’Brot A., Lu W.-J., Kurtz P., Moran J.V., Rakheja D., Chen K.S., Hammer R.E., Comerford S.A. et al. p53 genes function to restrain mobile elements. Genes Dev. 2016; 30:64–77. 26701264 10.1101/gad.266098.115 PMC4701979

[10] Rosenbloom K.R. , Armstrong J., Barber G.P., Casper J., Clawson H., Diekhans M., Dreszer T.R., Fujita P.A., Guruvadoo L., Haeussler M. et al. The UCSC genome browser database: 2015 update. Nucleic Acids Res. 2015; 43:D670–D681. 25428374 10.1093/nar/gku1177 PMC4383971

[11] Deininger P.L. , Jolly D.J., Rubin C.M., Friedmann T., Schmid C.W. Base sequence studies of 300 nucleotide renatured repeated human DNA clones. J. Mol. Biol. 1981; 151:17–33. 6276559 10.1016/0022-2836(81)90219-9

[12] Bao W. , Kojima K.K., Kohany O. Repbase update, a database of repetitive elements in eukaryotic genomes. Mob. DNA. 2015; 6:11. 26045719 10.1186/s13100-015-0041-9 PMC4455052

[13] Storer J. , Hubley R., Rosen J., Wheeler T.J., Smit A.F. The dfam community resource of transposable element families, sequence models, and genome annotations. Mob. DNA. 2021; 12:2. 33436076 10.1186/s13100-020-00230-y PMC7805219

[14] Hubley R. , Finn R.D., Clements J., Eddy S.R., Jones T.A., Bao W., Smit A.F.A., Wheeler T.J. The dfam database of repetitive DNA families. Nucleic Acids Res. 2016; 44:D81–D89. 26612867 10.1093/nar/gkv1272 PMC4702899

[15] Wheeler T.J. , Clements J., Eddy S.R., Hubley R., Jones T.a., Jurka J., Smit A.F., Finn R.D. Dfam: a database of repetitive DNA based on profile hidden markov models. Nucleic Acids Res. 2013; 41:D70–D82. 23203985 10.1093/nar/gks1265 PMC3531169

[16] Wang L. , Jiang T. On the complexity of multiple sequence alignment. J. Comput. Biol. 1994; 1:337–348. 8790475 10.1089/cmb.1994.1.337

[17] Notredame C. Recent evolutions of multiple sequence alignment algorithms. PLoS Comput. Biol. 2007; 3:e123. 17784778 10.1371/journal.pcbi.0030123 PMC1963500

[18] Sievers F. , Wilm A., Dineen D., Gibson T.J., Karplus K., Li W., Lopez R., McWilliam H., Remmert M., Söding J. et al. Fast, scalable generation of high-quality protein multiple sequence alignments using clustal omega. Mol. Syst. Biol. 2011; 7:539. 21988835 10.1038/msb.2011.75 PMC3261699

[19] Edgar R.C. MUSCLE: multiple sequence alignment with high accuracy and high throughput. Nucleic Acids Res. 2004; 32:1792–1797. 15034147 10.1093/nar/gkh340 PMC390337

[20] Lassmann T. , Frings O., Sonnhammer E.L.L. Kalign2: high-performance multiple alignment of protein and nucleotide sequences allowing external features. Nucleic Acids Res. 2009; 37:858–865. 19103665 10.1093/nar/gkn1006 PMC2647288

[21] Subramanian A.R. , Kaufmann M., Morgenstern B. DIALIGN-TX: greedy and progressive approaches for segment-based multiple sequence alignment. Algorithms Mol. Biol. 2008; 3:6. 18505568 10.1186/1748-7188-3-6 PMC2430965

[22] Notredame C. , Higgins D.G., Heringa J. T-Coffee: a novel method for fast and accurate multiple sequence alignment. J. Mol. Biol. 2000; 302:205–217. 10964570 10.1006/jmbi.2000.4042

[23] Do C.B. , Mahabhashyam M.S.P., Brudno M., Batzoglou S. ProbCons: probabilistic consistency-based multiple sequence alignment. Genome Res. 2005; 15:330–340. 15687296 10.1101/gr.2821705 PMC546535

[24] Katoh K. , Standley D.M. MAFFT multiple sequence alignment software version 7: improvements in performance and usability. Mol. Biol. Evol. 2013; 30:772–780. 23329690 10.1093/molbev/mst010 PMC3603318

[25] Flynn J.M. , Hubley R., Goubert C., Rosen J., Clark A.G., Feschotte C., Smit A.F. RepeatModeler2 for automated genomic discovery of transposable element families. Proc. Natl. Acad. Sci. U.S.A. 2020; 117:9451–9457. 32300014 10.1073/pnas.1921046117 PMC7196820

[26] Thompson J.D. , Linard B., Lecompte O., Poch O. A comprehensive benchmark study of multiple sequence alignment methods: current challenges and future perspectives. PLoS One. 2011; 6:e18093. 21483869 10.1371/journal.pone.0018093 PMC3069049

[27] Pais F.S.-M. , Ruy P.deC., Oliveira G., Coimbra R.S. Assessing the efficiency of multiple sequence alignment programs. Algorithms Mol. Biol. 2014; 9:4. 24602402 10.1186/1748-7188-9-4 PMC4015676

[28] Nute M. , Saleh E., Warnow T. Evaluating statistical multiple sequence alignment in comparison to other alignment methods on protein data sets. Syst. Biol. 2019; 68:396–411. 30329135 10.1093/sysbio/syy068 PMC6472439

[29] Aniba M.R. , Poch O., Thompson J.D. Issues in bioinformatics benchmarking: the case study of multiple sequence alignment. Nucleic Acids Res. 2010; 38:7353–7363. 20639539 10.1093/nar/gkq625 PMC2995051

[30] Bahr A. , Thompson J.D., Thierry J.-C., Poch O. BAliBASE (Benchmark alignment dataBASE): enhancements for repeats, transmembrane sequences and circular permutations. Nucleic Acids Res. 2001; 29:323–326. 11125126 10.1093/nar/29.1.323 PMC29792

[31] Van Walle I. , Lasters I., Wyns L. SABmark—a benchmark for sequence alignment that covers the entire known fold space. Bioinformatics. 2004; 21:1267–1268. 15333456 10.1093/bioinformatics/bth493

[32] Blackshields G. , Sievers F., Shi W., Wilm A., Higgins D.G. Sequence embedding for fast construction of guide trees for multiple sequence alignment. Algorithms Mol. Biol. 2010; 5:21. 20470396 10.1186/1748-7188-5-21 PMC2893182

[33] Mizuguchi K. , Deane C.M., Blundell T.L., Overington J.P. HOMSTRAD: a database of protein structure alignments for homologous families. Protein Sci. 1998; 7:2469–2471. 9828015 10.1002/pro.5560071126 PMC2143859

[34] Raghava G.P.S. , Searle S.M.J., Audley P.C., Barber J.D., Barton G.J. OXBench: a benchmark for evaluation of protein multiple sequence alignment accuracy. BMC Bioinf. 2003; 4:47. 10.1186/1471-2105-4-47 PMC280650 14552658

[35] Gardner P.P. , Wilm A., Washietl S. A benchmark of multiple sequence alignment programs upon structural RNAs. Nucleic Acids Res. 2005; 33:2433–2439. 15860779 10.1093/nar/gki541 PMC1087786

[36] Pollard D.A. , Bergman C.M., Stoye J., Celniker S.E., Eisen M.B. Benchmarking tools for the alignment of functional noncoding DNA. BMC Bioinf. 2004; 5:6. 10.1186/1471-2105-5-6 PMC344529 14736341

[37] Pervez M.T. , Babar M.E., Nadeem A., Aslam M., Awan A.R., Aslam N., Hussain T., Naveed N., Qadri S., Waheed U. et al. Evaluating the accuracy and efficiency of multiple sequence alignment methods. Evol. Bioinform. Online. 2014; 10:205–217. 25574120 10.4137/EBO.S19199 PMC4267518

[38] Pervez M.T. , Shah H.A., Babar M.E., Naveed N., Shoaib M. SAliBASE: a database of simulated protein alignments. Evol. Bioinform. Online. 2019; 15:1176934318821080. 30733625 10.1177/1176934318821080 PMC6343434

[39] Liu K. , Nelesen S., Raghavan S., Linder C.R., Warnow T. Barking up the wrong treelength: the impact of gap penalty on alignment and tree accuracy. IEEE/ACM Trans. Comput. Biol. Bioinform. 2009; 6:7–21. 19179695 10.1109/TCBB.2008.63

[40] Bull J.J. , Cunningham C.W., Molineux I.J., Badgett M.R., Hillis D.M. Experimental molecular evolution of bacteriophage t7. Evolution. 1993; 47:993–1007. 28564289 10.1111/j.1558-5646.1993.tb02130.x

[41] Garland T. , Dickerman A.W., Janis C.M., Jones J.A. Phylogenetic analysis of covariance by computer simulation. Syst. Biol. 1993; 42:265–292.

[42] Rambaut A. , Grassly N.C. Seq-Gen: an application for the monte carlo simulation of DNA sequence evolution along phylogenetic trees. Comput. Appl. Biosci. 1997; 13:235–238. 9183526 10.1093/bioinformatics/13.3.235

[43] Yang Z. PAML: a program package for phylogenetic analysis by maximum likelihood. Comput. Appl. Biosci. 1997; 13:555–556. 9367129 10.1093/bioinformatics/13.5.555

[44] Stoye J. , Evers D., Meyer F. Rose: generating sequence families. Bioinformatics. 1998; 14:157–163. 9545448 10.1093/bioinformatics/14.2.157

[45] Jermiin L.S. , Ho S.Y.W., Ababneh F., Robinson J., Larkum A.W.D. Hetero: a program to simulate the evolution of DNA on a four-taxon tree. Appl. Bioinformatics. 2003; 2:159–163. 15130802

[46] Rosenberg M.S. MySSP: Non-stationary evolutionary sequence simulation, including indels. Evol. Bioinform. Online. 2005; 1:117693430500100007. PMC2658873 19325855

[47] Cartwright R.A. DNA assembly with gaps (Dawg): simulating sequence evolution. Bioinformatics. 2005; 21:iii31–8. 16306390 10.1093/bioinformatics/bti1200

[48] Pang A. , Smith A.D., Nuin P.A.S., Tillier E.R.M. SIMPROT: using an empirically determined indel distribution in simulations of protein evolution. BMC Bioinf. 2005; 6:236. 10.1186/1471-2105-6-236 PMC1261159 16188037

[49] Strope C.L. , Scott S.D., Moriyama E.N. indel-Seq-Gen: a new protein family simulator incorporating domains, motifs, and indels. Mol. Biol. Evol. 2007; 24:640–649. 17158778 10.1093/molbev/msl195

[50] Fletcher W. , Yang Z. INDELible: a flexible simulator of biological sequence evolution. Mol. Biol. Evol. 2009; 26:1879–1888. 19423664 10.1093/molbev/msp098 PMC2712615

[51] Edgar R.C. 2009; Evolver.

[52] Nelson C.W. , Fu Y., Li W.-H. Trevolver: simulating non-reversible DNA sequence evolution in trinucleotide context on a bifurcating tree. 2019; bioRxiv doi:18 June 2019, preprint: not peer reviewed https://www.biorxiv.org/content/10.1101/672717v1.

[53] Thompson J.D. , Plewniak F., Poch O. A comprehensive comparison of multiple sequence alignment programs. Nucleic Acids Res. 1999; 27:2682–2690. 10373585 10.1093/nar/27.13.2682 PMC148477

[54] Michael Sauder J. , Arthur J.W., Dunbrack R.L. Large-Scale comparison of protein sequence alignment algorithms with structure alignments. Proteins Struct. Funct. Genet. 2000; 40:6–22. 10813826 10.1002/(sici)1097-0134(20000701)40:1<6::aid-prot30>3.0.co;2-7

[55] Schwartz A.S. , Myers E.W., Pachter L. Alignment Metric Accuracy. 2005; arXiv doi:27 October 2005, preprint: not peer reviewed https://arxiv.org/abs/q-bio/0510052.

[56] Edgar R.C. Quality measures for protein alignment benchmarks. Nucleic Acids Res. 2010; 38:2145–2153. 20047958 10.1093/nar/gkp1196 PMC2853116

[57] Siepel A. , Haussler D. Phylogenetic estimation of context-dependent substitution rates by maximum likelihood. Mol. Biol. Evol. 2004; 21:468–488. 14660683 10.1093/molbev/msh039

[58] Needleman S.B. , Wunsch C.D. A general method applicable to the search for similarities in the amino acid sequence of two proteins. J. Mol. Biol. 1970; 48:443–453. 5420325 10.1016/0022-2836(70)90057-4

[59] Hubley R.M. , Smit Arian A.F. 2010; RMBlast.

[60] Gish W. 1996; AB-BLAST.

[61] Storer J.M. , Hubley R., Rosen J., Smit A.F.A. Curation guidelines for de novo generated transposable element families. Curr Protoc. 2021; 1:e154. 34138525 10.1002/cpz1.154 PMC9191830

[62] Robertson H.M. , Martos R. Molecular evolution of the second ancient human mariner transposon, hsmar2, illustrates patterns of neutral evolution in the human genome lineage. Gene. 1997; 205:219–228. 9461396 10.1016/s0378-1119(97)00471-x

[63] Witherspoon D.J. , Robertson H.M. Neutral evolution of ten types of mariner transposons in the genomes of *Caenorhabditis elegans* and *Caenorhabditis briggsae*. J. Mol. Evol. 2003; 56:751–769. 12911038 10.1007/s00239-002-2450-x

[64] de Boer J.G. , Yazawa R., Davidson W.S., Koop B.F. Bursts and horizontal evolution of DNA transposons in the speciation of pseudotetraploid salmonids. BMC Genomics. 2007; 8:422. 18021408 10.1186/1471-2164-8-422 PMC2198921

[65] Hardies S.C. , Martin S.L., Voliva C.F., Hutchison C.A., Edgell M.H. An analysis of replacement and synonymous changes in the rodent L1 repeat family. Mol. Biol. Evol. 1986; 3:109–125. 3444397 10.1093/oxfordjournals.molbev.a040386

[66] Clough J.E. , Foster J.A., Barnett M., Wichman H.A. Computer simulation of transposable element evolution: random template and strict master models. J. Mol. Evol. 1996; 42:52–58. 8576964 10.1007/BF00163211

[67] Adey N.B. , Schichman S.A., Graham D.K., Peterson S.N., Edgell M.H., Hutchison C.A. 3rd Rodent L1 evolution has been driven by a single dominant lineage that has repeatedly acquired new transcriptional regulatory sequences. Mol. Biol. Evol. 1994; 11:778–789. 7968491 10.1093/oxfordjournals.molbev.a040158

[68] Smit A.F. , Riggs A.D. Tiggers and DNA transposon fossils in the human genome. Proc. Natl. Acad. Sci. U.S.A. 1996; 93:1443–1448. 8643651 10.1073/pnas.93.4.1443 PMC39958

[69] Kimura M. A simple method for estimating evolutionary rates of base substitutions through comparative studies of nucleotide sequences. J. Mol. Evol. 1980; 16:111–120. 7463489 10.1007/BF01731581

[70] Kruskal W.H. , Allen Wallis W. Use of ranks in one-criterion variance analysis. J. Am. Statist. Assoc. 1952; 47:583–621.

[71] Wilcoxon F. Individual comparisons by ranking methods. Biometrics Bull. 1945; 1:80–83.

[72] Smit A.F.A. Structure and evolution of mammalian interspersed repeats. 1996; University of Southern California.

[73] Robertson H.M. , Zumpano K.L. Molecular evolution of an ancient mariner transposon, hsmar1, in the human genome. Gene. 1997; 205:203–217. 9461395 10.1016/s0378-1119(97)00472-1

[74] Smit A. 2000; Dfam Family Zaphod (DF0001123) https://www.dfam.org/family/DF0001123/summary.

[75] Smit A. 2012; Dfam Family Zaphod2 (DF0001124) https://www.dfam.org/family/DF0001124.

[76] Smit A. 2008; Dfam Family Tigger10 (DF0000821). RepbaseUpdate https://www.dfam.org/family/DF0000821/summary.

[77] Smit A. 2012; Dfam Family Arthur2 (DF0001276) https://www.dfam.org/family/DF0001276/summary.

[78] Zoonomia Consortium A comparative genomics multitool for scientific discovery and conservation. Nature. 2020; 587:240–245. 33177664 10.1038/s41586-020-2876-6 PMC7759459

[79] Löytynoja A. , Goldman N. An algorithm for progressive multiple alignment of sequences with insertions. Proc. Natl. Acad. Sci. U.S.A. 2005; 102:10557–10562. 16000407 10.1073/pnas.0409137102 PMC1180752

[80] Di Tommaso P. , Chatzou M., Floden E.W., Barja P.P., Palumbo E., Notredame C. Nextflow enables reproducible computational workflows. Nat. Biotechnol. 2017; 35:316–319. 28398311 10.1038/nbt.3820

