# Supplementary material for: Accuracy of multiple sequence alignment methods in the reconstruction of transposable element families
Source: NAR Genom Bioinform. 2022 May 17;4(2):lqac040. doi: 10.1093/nargab/lqac040 (PMC9112768; doi:10.1093/nargab/lqac040)
Supplement: lqac040_Supplemental_Files [file lqac040_supplemental_files.zip › Supplemental - S1 - Revision 4.pdf]

S1 Kruskal-Wallis One Way Analysis of Variance and Wilcoxon Signed Rank Post-hoc Tests

S1.1 Sum of Pairs (SPS) Metrics

For each MSA tool evaluated, the full set of 180 SPS measurements (10 replicates, 18 divergence bins) were collected. A Kruskal-Wallis H-test was performed to assess if there is a significant difference in the accuracy of alignments between each pair of MSA tools. Additionally, a Wilcoxon signed rank post-hoc test was performed to generate multiple comparison tables for all pairs of methods. Section S1.1.1 contains the tables for the data presented in the main paper while section S1.1.2 includes tables for additional tree simulations, and additional seed sequences.

S1.1.1 Statistics for Main Paper Data

SPS Statistics for Tigger1 Sequence Divergence Analsysis
- Calculated on all replicate-parameter values

Tree Simulation: DNA Transposon Tree #1
Data File: paper-data/DNATransTree-1-Tigger1-R3S-eval/replicates.csv

Kruskal-Wallis H-test
H = 120.5978835 p = 2.49E-22

Wilcoxon signed rank test: mean_diff [p-val]

	refiner	muscle	mafft	dialign	kalign	fsa	clustalo	tcoffee	probcons
refiner		0.19 [7.0e-31]	-0.02 [7.6e-02]	0.12 [7.8e-30]	0.19 [7.8e-29]	0.03 [8.6e-06]	0.28 [1.2e-30]	0.18 [6.7e-31]	0.14 [3.2e-27]
muscle	-0.19 [7.0e-31]		-0.21 [2.7e-31]	-0.07 [1.9e-29]	-0.00 [1.5e-01]	-0.16 [6.8e-31]	0.09 [4.1e-20]	-0.02 [1.6e-07]	-0.05 [1.8e-09]
mafft	0.02 [7.6e-02]	0.21 [2.7e-31]		0.14 [1.2e-30]	0.21 [5.0e-29]	0.06 [1.8e-02]	0.30 [2.8e-31]	0.20 [2.7e-31]	0.16 [1.9e-14]
dialign	-0.12 [7.8e-30]	0.07 [1.9e-29]	-0.14 [1.2e-30]		0.07 [8.1e-10]	-0.08 [4.3e-17]	0.16 [3.3e-30]	0.06 [2.6e-17]	0.02 [6.9e-01]
kalign	-0.19 [7.8e-29]	0.00 [1.5e-01]	-0.21 [5.0e-29]	-0.07 [8.1e-10]		-0.16 [9.3e-28]	0.09 [2.4e-15]	-0.01 [2.7e-04]	-0.05 [4.9e-09]
fsa	-0.03 [8.6e-06]	0.16 [6.8e-31]	-0.06 [1.8e-02]	0.08 [4.3e-17]	0.16 [9.3e-28]		0.24 [6.8e-29]	0.14 [4.3e-30]	0.10 [6.0e-31]
clustalo	-0.28 [1.2e-30]	-0.09 [4.1e-20]	-0.30 [2.8e-31]	-0.16 [3.3e-30]	-0.09 [2.4e-15]	-0.24 [6.8e-29]		-0.10 [7.9e-25]	-0.14 [1.5e-17]
tcoffee	-0.18 [6.7e-31]	0.02 [1.6e-07]	-0.20 [2.7e-31]	-0.06 [2.6e-17]	0.01 [2.7e-04]	-0.14 [4.3e-30]	0.10 [7.9e-25]		-0.04 [1.4e-07]
probcons	-0.14 [3.2e-27]	0.05 [1.8e-09]	-0.16 [1.9e-14]	-0.02 [6.9e-01]	0.05 [4.9e-09]	-0.10 [6.0e-31]	0.14 [1.5e-17]	0.04 [1.4e-07]	

SPS Statistics for L2 Sequence Divergence Analsysis
- Calculated on all replicate-parameter values

Tree Simulation: LINE Tree #1
Data File: paper-data/LINETree-1-L2-R3S-eval/replicates.csv

Kruskal-Wallis H-test

	H =	154.7677666	p =	1.98E-29

Wilcoxon signed rank test: mean_diff [p-val]

	refiner	muscle	mafft	dialign	kalign	fsa	clustalo	tcoffee	probcons
refiner		0.19 [2.7e-31]	-0.01 [1.3e-02]	0.11 [1.8e-30]	0.21 [9.7e-31]	0.04 [2.1e-08]	0.26 [2.7e-31]	0.17 [2.7e-31]	0.16 [2.9e-30]
muscle	-0.19 [2.7e-31]		-0.21 [2.7e-31]	-0.08 [4.8e-31]	0.02 [2.7e-02]	-0.16 [2.7e-31]	0.07 [3.2e-19]	-0.03 [5.5e-13]	-0.03 [7.6e-05]
mafft	0.01 [1.3e-02]	0.21 [2.7e-31]		0.13 [5.8e-31]	0.23 [2.6e-30]	0.05 [8.7e-04]	0.28 [2.7e-31]	0.18 [2.7e-31]	0.18 [1.4e-21]
dialign	-0.11 [1.8e-30]	0.08 [4.8e-31]	-0.13 [5.8e-31]		0.10 [1.4e-25]	-0.08 [2.5e-21]	0.15 [2.7e-31]	0.06 [7.9e-26]	0.05 [8.1e-04]
kalign	-0.21 [9.7e-31]	-0.02 [2.7e-02]	-0.23 [2.6e-30]	-0.10 [1.4e-25]		-0.18 [1.8e-30]	0.05 [7.8e-09]	-0.04 [2.5e-18]	-0.05 [1.1e-10]
fsa	-0.04 [2.1e-08]	0.16 [2.7e-31]	-0.05 [8.7e-04]	0.08 [2.5e-21]	0.18 [1.8e-30]		0.23 [2.9e-31]	0.13 [2.7e-31]	0.13 [1.3e-30]
clustalo	-0.26 [2.7e-31]	-0.07 [3.2e-19]	-0.28 [2.7e-31]	-0.15 [2.7e-31]	-0.05 [7.8e-09]	-0.23 [2.9e-31]		-0.09 [1.7e-24]	-0.10 [5.1e-14]
tcoffee	-0.17 [2.7e-31]	0.03 [5.5e-13]	-0.18 [2.7e-31]	-0.06 [7.9e-26]	0.04 [2.5e-18]	-0.13 [2.7e-31]	0.09 [1.7e-24]		-0.01 [1.2e-01]
probcons	-0.16 [2.9e-30]	0.03 [7.6e-05]	-0.18 [1.4e-21]	-0.05 [8.1e-04]	0.05 [1.1e-10]	-0.13 [1.3e-30]	0.10 [5.1e-14]	0.01 [1.2e-01]	

SPS Statistics for Tigger1 Sequence Fragmentation Analysis
- Calculated on all replicate-parameter values

Tree Simulation: DNA Transposon Tree #1
Fragmentation Simulation: Low Divergence Sequences [gput100]
Data File: paper-data/DNATransTree-1-Tigger1-R3S-gput100-mfl2-eval/replicates.csv

Kruskal-Wallis H-test

	H =	633.1497984	p =	1.74E-131

Wilcoxon signed rank test: mean_diff [p-val]

	refiner	muscle	mafft	dialign	kalign	fsa	clustalo	tcoffee	probcons
refiner		0.32 [2.0e-21]	0.17 [8.9e-17]	0.00 [1.9e-14]	0.04 [4.6e-19]	0.01 [2.5e-16]	0.05 [2.0e-21]	0.29 [2.0e-21]	0.46 [2.0e-21]
muscle	-0.32 [2.0e-21]		-0.15 [2.8e-13]	-0.32 [2.9e-21]	-0.28 [1.6e-20]	-0.31 [2.0e-21]	-0.27 [8.0e-17]	-0.03 [3.3e-02]	0.14 [2.5e-04]
mafft	-0.17 [8.9e-17]	0.15 [2.8e-13]		-0.17 [6.2e-14]	-0.13 [3.2e-13]	-0.16 [1.6e-15]	-0.12 [1.0e-09]	0.12 [9.3e-17]	0.29 [1.8e-19]
dialign	-0.00 [1.9e-14]	0.32 [2.9e-21]	0.17 [6.2e-14]		0.04 [8.7e-15]	0.01 [8.3e-12]	0.05 [2.0e-21]	0.29 [2.0e-21]	0.45 [2.6e-21]
kalign	-0.04 [4.6e-19]	0.28 [1.6e-20]	0.13 [3.2e-13]	-0.04 [8.7e-15]		-0.03 [6.8e-11]	0.01 [2.3e-02]	0.25 [2.1e-21]	0.42 [6.2e-21]
fsa	-0.01 [2.5e-16]	0.31 [2.0e-21]	0.16 [1.6e-15]	-0.01 [8.3e-12]	0.03 [6.8e-11]		0.04 [1.4e-20]	0.28 [2.0e-21]	0.45 [2.0e-21]
clustalo	-0.05 [2.0e-21]	0.27 [8.0e-17]	0.12 [1.0e-09]	-0.05 [2.0e-21]	-0.01 [2.3e-02]	-0.04 [1.4e-20]		0.24 [5.6e-15]	0.40 [8.2e-12]
tcoffee	-0.29 [2.0e-21]	0.03 [3.3e-02]	-0.12 [9.3e-17]	-0.29 [2.0e-21]	-0.25 [2.1e-21]	-0.28 [2.0e-21]	-0.24 [5.6e-15]		0.16 [6.3e-09]
probcons	-0.46 [2.0e-21]	-0.14 [2.5e-04]	-0.29 [1.8e-19]	-0.45 [2.6e-21]	-0.42 [6.2e-21]	-0.45 [2.0e-21]	-0.40 [8.2e-12]	-0.16 [6.3e-09]	

SPS Statistics for Tigger1 Sequence Fragmentation Analysis
- Calculated on all replicate-parameter values

Tree Simulation: DNA Transposon Tree #1
Fragmentation Simulation: High Divergence Sequences [gput3000]
Data File: paper-data/DNATransTree-1-Tigger1-R3S-gput3000-mfl2-eval/replicates.csv

Kruskal-Wallis H-test

H = 936.5867551 p = 7.23E-197

Wilcoxon signed rank test: mean_diff [p-val]

	refiner	muscle	mafft	dialign	kalign	fsa	clustalo	tcoffee	probcons
refiner		0.46 [2.0e-21]	0.11 [1.1e-11]	0.11 [2.0e-20]	0.43 [2.0e-21]	0.13 [3.8e-21]	0.39 [2.0e-21]	0.48 [2.0e-21]	0.48 [2.0e-21]
muscle	-0.46 [2.0e-21]		-0.35 [2.0e-21]	-0.35 [2.0e-21]	-0.03 [1.7e-12]	-0.33 [2.0e-21]	-0.07 [1.0e-12]	0.02 [1.3e-02]	0.02 [1.7e-18]
mafft	-0.11 [1.1e-11]	0.35 [2.0e-21]		-0.01 [5.3e-01]	0.31 [2.0e-21]	0.01 [3.5e-01]	0.28 [5.4e-21]	0.36 [2.0e-21]	0.37 [2.0e-21]
dialign	-0.11 [2.0e-20]	0.35 [2.0e-21]	0.01 [5.3e-01]		0.32 [2.0e-21]	0.02 [4.5e-05]	0.29 [2.0e-21]	0.37 [2.0e-21]	0.38 [2.0e-21]
kalign	-0.43 [2.0e-21]	0.03 [1.7e-12]	-0.31 [2.0e-21]	-0.32 [2.0e-21]		-0.30 [2.0e-21]	-0.04 [1.1e-11]	0.05 [2.0e-21]	0.05 [2.0e-21]
fsa	-0.13 [3.8e-21]	0.33 [2.0e-21]	-0.01 [3.5e-01]	-0.02 [4.5e-05]	0.30 [2.0e-21]		0.27 [2.0e-21]	0.35 [2.0e-21]	0.36 [2.0e-21]
clustalo	-0.39 [2.0e-21]	0.07 [1.0e-12]	-0.28 [5.4e-21]	-0.29 [2.0e-21]	0.04 [1.1e-11]	-0.27 [2.0e-21]		0.08 [2.7e-21]	0.09 [2.2e-21]
tcoffee	-0.48 [2.0e-21]	-0.02 [1.3e-02]	-0.36 [2.0e-21]	-0.37 [2.0e-21]	-0.05 [2.0e-21]	-0.35 [2.0e-21]	-0.08 [2.7e-21]		0.01 [1.8e-19]
probcons	-0.48 [2.0e-21]	-0.02 [1.7e-18]	-0.37 [2.0e-21]	-0.38 [2.0e-21]	-0.05 [2.0e-21]	-0.36 [2.0e-21]	-0.09 [2.2e-21]	-0.01 [1.8e-19]	

S1.1.2 Statistics for Additional Trees and Seed Sequences

SPS Statistics for Charlie1 Sequence Divergence Analsysis
- Calculated on all replicate-parameter values

Tree Simulation: DNA Transposon Tree #1
Data File: paper-data/DNATransTree-1-Charlie1-R3S-eval/replicates.csv

Kruskal-Wallis H-test

H = 121.3730795 p = 1.73E-22

Wilcoxon signed rank test: mean_diff [p-val]

	refiner	muscle	mafft	dialign	kalign	fsa	clustalo	tcoffee	probcons
refiner		0.21 [8.1e-31]	-0.02 [2.9e-03]	0.13 [6.8e-30]	0.20 [1.2e-29]	0.04 [1.5e-05]	0.28 [2.3e-30]	0.18 [8.6e-31]	0.16 [2.0e-29]
muscle	-0.21 [8.1e-31]		-0.23 [2.7e-31]	-0.08 [1.4e-28]	-0.01 [1.8e-02]	-0.16 [7.7e-31]	0.07 [6.7e-15]	-0.02 [4.1e-09]	-0.05 [2.8e-08]
mafft	0.02 [2.9e-03]	0.23 [2.7e-31]		0.15 [4.0e-31]	0.22 [1.8e-29]	0.07 [1.9e-03]	0.30 [3.2e-31]	0.21 [2.7e-31]	0.18 [7.2e-18]
dialign	-0.13 [6.8e-30]	0.08 [1.4e-28]	-0.15 [4.0e-31]		0.07 [4.2e-12]	-0.08 [2.1e-15]	0.16 [1.9e-30]	0.06 [3.3e-14]	0.03 [2.5e-01]
kalign	-0.20 [1.2e-29]	0.01 [1.8e-02]	-0.22 [1.8e-29]	-0.07 [4.2e-12]		-0.16 [3.6e-28]	0.08 [2.7e-13]	-0.02 [8.6e-06]	-0.04 [5.6e-06]
fsa	-0.04 [1.5e-05]	0.16 [7.7e-31]	-0.07 [1.9e-03]	0.08 [2.1e-15]	0.16 [3.6e-28]		0.24 [3.1e-29]	0.14 [5.7e-30]	0.12 [5.8e-31]
clustalo	-0.28 [2.3e-30]	-0.07 [6.7e-15]	-0.30 [3.2e-31]	-0.16 [1.9e-30]	-0.08 [2.7e-13]	-0.24 [3.1e-29]		-0.10 [3.8e-22]	-0.12 [1.2e-13]
tcoffee	-0.18 [8.6e-31]	0.02 [4.1e-09]	-0.21 [2.7e-31]	-0.06 [3.3e-14]	0.02 [8.6e-06]	-0.14 [5.7e-30]	0.10 [3.8e-22]		-0.02 [4.0e-04]
probcons	-0.16 [2.0e-29]	0.05 [2.8e-08]	-0.18 [7.2e-18]	-0.03 [2.5e-01]	0.04 [5.6e-06]	-0.12 [5.8e-31]	0.12 [1.2e-13]	0.02 [4.0e-04]	

SPS Statistics for CR1 Sequence Divergence Analsysis

- Calculated on all replicate-parameter values

Tree Simulation: LINE Tree #1
Data File: paper-data/LINETree-1-CR1-R3S-eval/replicates.csv

Kruskal-Wallis H-test

H =	153.8019521	p =	3.16E-29

Wilcoxon signed rank test: mean_diff [p-val]

	refiner	muscle	mafft	dialign	kalign	fsa	clustalo	tcoffee	probcons
refiner		0.18 [2.7e-31]	-0.01 [7.2e-01]	0.11 [8.9e-31]	0.18 [1.2e-30]	0.03 [1.9e-06]	0.26 [2.7e-31]	0.16 [2.7e-31]	0.11 [8.6e-25]
muscle	-0.18 [2.7e-31]		-0.20 [2.7e-31]	-0.08 [3.3e-31]	-0.00 [1.4e-01]	-0.16 [2.7e-31]	0.07 [3.5e-19]	-0.02 [1.2e-09]	-0.07 [4.6e-16]
mafft	0.01 [7.2e-01]	0.20 [2.7e-31]		0.12 [2.6e-30]	0.19 [2.0e-29]	0.04 [3.4e-02]	0.27 [2.7e-31]	0.17 [2.7e-31]	0.12 [6.9e-12]
dialign	-0.11 [8.9e-31]	0.08 [3.3e-31]	-0.12 [2.6e-30]		0.07 [2.0e-12]	-0.08 [3.9e-22]	0.15 [2.7e-31]	0.05 [4.4e-25]	0.01 [4.4e-01]
kalign	-0.18 [1.2e-30]	0.00 [1.4e-01]	-0.19 [2.0e-29]	-0.07 [2.0e-12]		-0.15 [3.5e-30]	0.07 [4.2e-12]	-0.02 [1.5e-03]	-0.07 [3.3e-17]
fsa	-0.03 [1.9e-06]	0.16 [2.7e-31]	-0.04 [3.4e-02]	0.08 [3.9e-22]	0.15 [3.5e-30]		0.23 [3.0e-31]	0.13 [2.7e-31]	0.08 [9.8e-30]
clustalo	-0.26 [2.7e-31]	-0.07 [3.5e-19]	-0.27 [2.7e-31]	-0.15 [2.7e-31]	-0.07 [4.2e-12]	-0.23 [3.0e-31]		-0.09 [1.4e-23]	-0.14 [1.6e-21]
tcoffee	-0.16 [2.7e-31]	0.02 [1.2e-09]	-0.17 [2.7e-31]	-0.05 [4.4e-25]	0.02 [1.5e-03]	-0.13 [2.7e-31]	0.09 [1.4e-23]		-0.05 [6.8e-13]
probcons	-0.11 [8.6e-25]	0.07 [4.6e-16]	-0.12 [6.9e-12]	-0.01 [4.4e-01]	0.07 [3.3e-17]	-0.08 [9.8e-30]	0.14 [1.6e-21]	0.05 [6.8e-13]	

SPS Statistics for L2 Sequence Fragmentation Analysis

- Calculated on all replicate-parameter values

Tree Simulation: LINE Tree #1
Fragmentation Simulation: Low Divergence Sequences [gput100]
Data File: paper-data/LINETree-1-L2-R3S-gput100-mfl2-eval/replicates.csv

Kruskal-Wallis H-test

H =	742.6302377	p =	4.73E-155

Wilcoxon signed rank test: mean_diff [p-val]

	refiner	muscle	mafft	dialign	kalign	fsa	clustalo	tcoffee	probcons
refiner		0.50 [2.0e-21]	0.21 [3.9e-18]	0.00 [2.2e-13]	0.13 [2.3e-20]	0.01 [5.7e-17]	0.04 [2.0e-21]	0.39 [2.0e-21]	0.60 [2.0e-21]
muscle	-0.50 [2.0e-21]		-0.28 [3.8e-21]	-0.50 [2.0e-21]	-0.37 [2.4e-21]	-0.49 [2.0e-21]	-0.46 [2.3e-21]	-0.11 [2.1e-12]	0.10 [8.3e-05]
mafft	-0.21 [3.9e-18]	0.28 [3.8e-21]		-0.21 [9.6e-16]	-0.09 [2.6e-07]	-0.20 [1.3e-16]	-0.17 [1.4e-12]	0.17 [3.3e-21]	0.38 [2.0e-21]
dialign	-0.00 [2.2e-13]	0.50 [2.0e-21]	0.21 [9.6e-16]		0.13 [1.4e-19]	0.01 [3.4e-14]	0.04 [2.0e-21]	0.39 [2.0e-21]	0.60 [2.0e-21]
kalign	-0.13 [2.3e-20]	0.37 [2.4e-21]	0.09 [2.6e-07]	-0.13 [1.4e-19]		-0.12 [7.3e-20]	-0.08 [1.0e-11]	0.26 [2.9e-20]	0.47 [1.9e-19]
fsa	-0.01 [5.7e-17]	0.49 [2.0e-21]	0.20 [1.3e-16]	-0.01 [3.4e-14]	0.12 [7.3e-20]		0.03 [2.1e-18]	0.38 [2.0e-21]	0.59 [2.0e-21]
clustalo	-0.04 [2.0e-21]	0.46 [2.3e-21]	0.17 [1.4e-12]	-0.04 [2.0e-21]	0.08 [1.0e-11]	-0.03 [2.1e-18]		0.34 [5.4e-19]	0.55 [6.0e-18]
tcoffee	-0.39 [2.0e-21]	0.11 [2.1e-12]	-0.17 [3.3e-21]	-0.39 [2.0e-21]	-0.26 [2.9e-20]	-0.38 [2.0e-21]	-0.34 [5.4e-19]		0.21 [3.3e-16]
probcons	-0.60 [2.0e-21]	-0.10 [8.3e-05]	-0.38 [2.0e-21]	-0.60 [2.0e-21]	-0.47 [1.9e-19]	-0.59 [2.0e-21]	-0.55 [6.0e-18]	-0.21 [3.3e-16]	

SPS Statistics for L2 Sequence Fragmentation Analysis

- Calculated on all replicate-parameter values

Tree Simulation: LINE Tree #1
Fragmentation Simulation: Medium Divergence Sequences [gput1500]
Data File: paper-data/LINETree-1-L2-R3S-gput1500-mfl2-eval/replicates.csv

Kruskal-Wallis H-test

H = 892.1579728 p = 2.78E-187

Wilcoxon signed rank test: mean_diff [p-val]

	refiner	muscle	mafft	dialign	kalign	fsa	clustalo	tcoffee	probcons
refiner		0.71 [2.0e-21]	0.10 [1.0e-08]	0.04 [6.3e-08]	0.36 [2.2e-21]	-0.05 [2.5e-07]	0.32 [2.0e-21]	0.63 [2.0e-21]	0.70 [2.0e-21]
muscle	-0.71 [2.0e-21]		-0.62 [2.0e-21]	-0.67 [2.0e-21]	-0.36 [2.0e-21]	-0.76 [2.0e-21]	-0.39 [3.0e-21]	-0.09 [5.0e-21]	-0.01 [1.4e-01]
mafft	-0.10 [1.0e-08]	0.62 [2.0e-21]		-0.06 [6.8e-05]	0.26 [2.0e-21]	-0.15 [2.0e-21]	0.22 [6.4e-20]	0.53 [2.0e-21]	0.60 [2.0e-21]
dialign	-0.04 [6.3e-08]	0.67 [2.0e-21]	0.06 [6.8e-05]		0.32 [2.6e-21]	-0.09 [2.1e-21]	0.28 [2.0e-21]	0.59 [2.0e-21]	0.66 [2.4e-21]
kalign	-0.36 [2.2e-21]	0.36 [2.0e-21]	-0.26 [2.0e-21]	-0.32 [2.6e-21]		-0.41 [2.0e-21]	-0.04 [8.5e-04]	0.27 [4.5e-21]	0.34 [1.2e-20]
fsa	0.05 [2.5e-07]	0.76 [2.0e-21]	0.15 [2.0e-21]	0.09 [2.1e-21]	0.41 [2.0e-21]		0.37 [2.0e-21]	0.68 [2.0e-21]	0.75 [2.0e-21]
clustalo	-0.32 [2.0e-21]	0.39 [3.0e-21]	-0.22 [6.4e-20]	-0.28 [2.0e-21]	0.04 [8.5e-04]	-0.37 [2.0e-21]		0.31 [2.1e-19]	0.38 [1.7e-19]
tcoffee	-0.63 [2.0e-21]	0.09 [5.0e-21]	-0.53 [2.0e-21]	-0.59 [2.0e-21]	-0.27 [4.5e-21]	-0.68 [2.0e-21]	-0.31 [2.1e-19]		0.07 [1.7e-15]
probcons	-0.70 [2.0e-21]	0.01 [1.4e-01]	-0.60 [2.0e-21]	-0.66 [2.4e-21]	-0.34 [1.2e-20]	-0.75 [2.0e-21]	-0.38 [1.7e-19]	-0.07 [1.7e-15]	

SPS Statistics for L2 Sequence Fragmentation Analysis

- Calculated on all replicate-parameter values

Tree Simulation: LINE Tree #1
Fragmentation Simulation: High Divergence Sequences [gput3000]
Data File: paper-data/LINETree-1-L2-R3S-gput3000-mfl2-eval/replicates.csv

Kruskal-Wallis H-test

H = 936.5036137 p = 7.53E-197

Wilcoxon signed rank test: mean_diff [p-val]

	refiner	muscle	mafft	dialign	kalign	fsa	clustalo	tcoffee	probcons
refiner		0.42 [2.0e-21]	0.08 [3.0e-06]	-0.01 [4.4e-01]	0.35 [2.0e-21]	-0.00 [5.8e-01]	0.29 [2.1e-21]	0.41 [2.0e-21]	0.44 [2.0e-21]
muscle	-0.42 [2.0e-21]		-0.34 [2.0e-21]	-0.43 [2.0e-21]	-0.07 [3.6e-19]	-0.42 [2.0e-21]	-0.13 [2.4e-21]	-0.01 [1.3e-09]	0.01 [2.3e-18]
mafft	-0.08 [3.0e-06]	0.34 [2.0e-21]		-0.09 [2.3e-08]	0.27 [2.0e-21]	-0.08 [1.3e-10]	0.21 [1.5e-19]	0.33 [2.0e-21]	0.36 [2.0e-21]
dialign	0.01 [4.4e-01]	0.43 [2.0e-21]	0.09 [2.3e-08]		0.36 [2.0e-21]	0.01 [6.7e-02]	0.30 [2.0e-21]	0.42 [2.0e-21]	0.44 [2.0e-21]
kalign	-0.35 [2.0e-21]	0.07 [3.6e-19]	-0.27 [2.0e-21]	-0.36 [2.0e-21]		-0.36 [2.0e-21]	-0.06 [7.6e-20]	0.06 [5.6e-21]	0.08 [2.0e-21]
fsa	0.00 [5.8e-01]	0.42 [2.0e-21]	0.08 [1.3e-10]	-0.01 [6.7e-02]	0.36 [2.0e-21]		0.29 [2.0e-21]	0.41 [2.0e-21]	0.44 [2.0e-21]
clustalo	-0.29 [2.1e-21]	0.13 [2.4e-21]	-0.21 [1.5e-19]	-0.30 [2.0e-21]	0.06 [7.6e-20]	-0.29 [2.0e-21]		0.12 [2.0e-21]	0.14 [2.0e-21]
tcoffee	-0.41 [2.0e-21]	0.01 [1.3e-09]	-0.33 [2.0e-21]	-0.42 [2.0e-21]	-0.06 [5.6e-21]	-0.41 [2.0e-21]	-0.12 [2.0e-21]		0.02 [2.0e-21]
probcons	-0.44 [2.0e-21]	-0.01 [2.3e-18]	-0.36 [2.0e-21]	-0.44 [2.0e-21]	-0.08 [2.0e-21]	-0.44 [2.0e-21]	-0.14 [2.0e-21]	-0.02 [2.0e-21]	

SPS Statistics for Tigger1 Sequence Fragmentation Analysis

- Calculated on all replicate-parameter values

Tree Simulation: DNA Transposon Tree #1
Fragmentation Simulation: Medium Divergence Sequences [gput1500]
Data File: paper-data/DNATransTree-1-Tigger1-R3S-gput1500-mfl2-eval/replicates.csv

Kruskal-Wallis H-test

H = 814.2835392 p = 1.72E-170

Wilcoxon signed rank test: mean_diff [p-val]

	refiner	muscle	mafft	dialign	kalign	fsa	clustalo	tcoffee	probcons
refiner		0.69 [2.0e-21]	0.12 [9.4e-20]	0.12 [2.0e-21]	0.37 [2.0e-21]	0.00 [4.1e-01]	0.41 [2.0e-21]	0.63 [2.0e-21]	0.64 [5.4e-21]
muscle	-0.69 [2.0e-21]		-0.57 [2.0e-21]	-0.57 [2.5e-21]	-0.32 [2.2e-21]	-0.69 [2.0e-21]	-0.28 [7.1e-18]	-0.06 [1.3e-16]	-0.05 [2.6e-02]
mafft	-0.12 [9.4e-20]	0.57 [2.0e-21]		-0.00 [6.7e-01]	0.24 [2.2e-21]	-0.12 [2.0e-21]	0.28 [4.9e-21]	0.51 [2.0e-21]	0.51 [4.1e-21]
dialign	-0.12 [2.0e-21]	0.57 [2.5e-21]	0.00 [6.7e-01]		0.25 [3.4e-20]	-0.12 [2.1e-21]	0.29 [2.0e-21]	0.51 [1.3e-20]	0.52 [3.3e-18]
kalign	-0.37 [2.0e-21]	0.32 [2.2e-21]	-0.24 [2.2e-21]	-0.25 [3.4e-20]		-0.37 [2.0e-21]	0.04 [2.3e-03]	0.26 [2.8e-20]	0.27 [7.9e-16]
fsa	-0.00 [4.1e-01]	0.69 [2.0e-21]	0.12 [2.0e-21]	0.12 [2.1e-21]	0.37 [2.0e-21]		0.40 [2.0e-21]	0.63 [2.0e-21]	0.63 [2.0e-21]
clustalo	-0.41 [2.0e-21]	0.28 [7.1e-18]	-0.28 [4.9e-21]	-0.29 [2.0e-21]	-0.04 [2.3e-03]	-0.40 [2.0e-21]		0.22 [5.4e-15]	0.23 [1.3e-11]
tcoffee	-0.63 [2.0e-21]	0.06 [1.3e-16]	-0.51 [2.0e-21]	-0.51 [1.3e-20]	-0.26 [2.8e-20]	-0.63 [2.0e-21]	-0.22 [5.4e-15]		0.01 [5.1e-01]
probcons	-0.64 [5.4e-21]	0.05 [2.6e-02]	-0.51 [4.1e-21]	-0.52 [3.3e-18]	-0.27 [7.9e-16]	-0.63 [2.0e-21]	-0.23 [1.3e-11]	-0.01 [5.1e-01]	

SPS Statistics for Tigger1 Sequence Divergence Analsysis

- Calculated on all replicate-parameter values

Tree Simulation: DNA Transposon Tree #2
Data File: paper-data/DNATransTree-2-Tigger1-R3S-eval/replicates.csv

Kruskal-Wallis H-test

H = 118.5481616 p = 6.61E-22

Wilcoxon signed rank test: mean_diff [p-val]

	refiner	muscle	mafft	dialign	kalign	fsa	clustalo	tcoffee	probcons
refiner		0.19 [1.0e-30]	-0.02 [9.3e-03]	0.12 [2.7e-29]	0.19 [4.0e-29]	0.04 [6.7e-06]	0.28 [3.0e-30]	0.18 [7.6e-31]	0.14 [3.6e-29]
muscle	-0.19 [1.0e-30]		-0.21 [4.0e-31]	-0.08 [9.3e-30]	-0.00 [9.3e-02]	-0.16 [2.1e-30]	0.09 [2.2e-19]	-0.01 [3.4e-05]	-0.05 [7.1e-09]
mafft	0.02 [9.3e-03]	0.21 [4.0e-31]		0.14 [1.2e-30]	0.21 [4.3e-29]	0.06 [1.3e-02]	0.30 [3.0e-31]	0.20 [2.7e-31]	0.16 [1.6e-14]
dialign	-0.12 [2.7e-29]	0.08 [9.3e-30]	-0.14 [1.2e-30]		0.07 [3.3e-10]	-0.08 [9.2e-16]	0.16 [3.2e-30]	0.06 [5.6e-19]	0.02 [7.3e-01]
kalign	-0.19 [4.0e-29]	0.00 [9.3e-02]	-0.21 [4.3e-29]	-0.07 [3.3e-10]		-0.15 [3.3e-28]	0.09 [1.9e-15]	-0.01 [6.3e-03]	-0.05 [1.5e-08]
fsa	-0.04 [6.7e-06]	0.16 [2.1e-30]	-0.06 [1.3e-02]	0.08 [9.2e-16]	0.15 [3.3e-28]		0.24 [4.0e-29]	0.14 [2.9e-30]	0.10 [2.6e-29]
clustalo	-0.28 [3.0e-30]	-0.09 [2.2e-19]	-0.30 [3.0e-31]	-0.16 [3.2e-30]	-0.09 [1.9e-15]	-0.24 [4.0e-29]		-0.10 [4.4e-24]	-0.14 [3.0e-17]
tcoffee	-0.18 [7.6e-31]	0.01 [3.4e-05]	-0.20 [2.7e-31]	-0.06 [5.6e-19]	0.01 [6.3e-03]	-0.14 [2.9e-30]	0.10 [4.4e-24]		-0.04 [8.8e-08]
probcons	-0.14 [3.6e-29]	0.05 [7.1e-09]	-0.16 [1.6e-14]	-0.02 [7.3e-01]	0.05 [1.5e-08]	-0.10 [2.6e-29]	0.14 [3.0e-17]	0.04 [8.8e-08]	

SPS Statistics for CR1 Sequence Divergence Analsysis

- Calculated on all replicate-parameter values

Tree Simulation: LINE Tree #2
Data File: paper-data/LINETree-2-CR1-R3S-eval/replicates.csv

Kruskal-Wallis H-test

	H =	167.9296916	p =	3.50E-32

Wilcoxon signed rank test: mean_diff [p-val]

	refiner	muscle	mafft	dialign	kalign	fsa	clustalo	tcoffee	probcons
refiner		0.13 [5.8e-31]	-0.03 [5.4e-10]	0.06 [6.3e-29]	0.12 [5.5e-30]	0.00 [5.0e-01]	0.19 [2.7e-31]	0.11 [2.7e-31]	0.05 [5.0e-23]
muscle	-0.13 [5.8e-31]		-0.16 [4.0e-31]	-0.08 [4.2e-31]	-0.01 [7.0e-04]	-0.13 [4.0e-31]	0.06 [2.7e-16]	-0.03 [3.5e-16]	-0.08 [7.3e-27]
mafft	0.03 [5.4e-10]	0.16 [4.0e-31]		0.09 [8.5e-31]	0.15 [1.6e-29]	0.03 [3.1e-03]	0.22 [2.7e-31]	0.14 [2.7e-31]	0.08 [5.1e-15]
dialign	-0.06 [6.3e-29]	0.08 [4.2e-31]	-0.09 [8.5e-31]		0.06 [2.9e-14]	-0.05 [2.1e-26]	0.14 [2.7e-31]	0.05 [2.8e-29]	-0.01 [7.4e-03]
kalign	-0.12 [5.5e-30]	0.01 [7.0e-04]	-0.15 [1.6e-29]	-0.06 [2.9e-14]		-0.12 [8.8e-30]	0.07 [1.2e-13]	-0.01 [2.1e-03]	-0.07 [1.8e-27]
fsa	-0.00 [5.0e-01]	0.13 [4.0e-31]	-0.03 [3.1e-03]	0.05 [2.1e-26]	0.12 [8.8e-30]		0.19 [2.9e-31]	0.10 [2.7e-31]	0.05 [9.7e-30]
clustalo	-0.19 [2.7e-31]	-0.06 [2.7e-16]	-0.22 [2.7e-31]	-0.14 [2.7e-31]	-0.07 [1.2e-13]	-0.19 [2.9e-31]		-0.09 [2.0e-23]	-0.14 [9.2e-26]
tcoffee	-0.11 [2.7e-31]	0.03 [3.5e-16]	-0.14 [2.7e-31]	-0.05 [2.8e-29]	0.01 [2.1e-03]	-0.10 [2.7e-31]	0.09 [2.0e-23]		-0.06 [9.4e-23]
probcons	-0.05 [5.0e-23]	0.08 [7.3e-27]	-0.08 [5.1e-15]	0.01 [7.4e-03]	0.07 [1.8e-27]	-0.05 [9.7e-30]	0.14 [9.2e-26]	0.06 [9.4e-23]	

SPS Statistics for L2 Sequence Divergence Analsysis

- Calculated on all replicate-parameter values

Tree Simulation: LINE Tree #2
Data File: paper-data/LINETree-2-L2-R3S-eval/replicates.csv

Kruskal-Wallis H-test

	H =	164.7108531	p =	1.65E-31

Wilcoxon signed rank test: mean_diff [p-val]

	refiner	muscle	mafft	dialign	kalign	fsa	clustalo	tcoffee	probcons
refiner		0.14 [2.8e-31]	-0.03 [1.0e-14]	0.06 [1.5e-29]	0.15 [2.7e-30]	0.01 [6.2e-03]	0.20 [2.7e-31]	0.11 [4.7e-31]	0.09 [2.7e-26]
muscle	-0.14 [2.8e-31]		-0.17 [4.0e-31]	-0.08 [2.9e-31]	0.01 [8.9e-02]	-0.13 [2.8e-31]	0.06 [2.1e-18]	-0.03 [4.2e-20]	-0.05 [4.5e-14]
mafft	0.03 [1.0e-14]	0.17 [4.0e-31]		0.09 [1.1e-30]	0.18 [7.7e-31]	0.04 [5.2e-06]	0.23 [2.7e-31]	0.14 [2.8e-31]	0.12 [4.2e-24]
dialign	-0.06 [1.5e-29]	0.08 [2.9e-31]	-0.09 [1.1e-30]		0.09 [1.3e-25]	-0.05 [7.6e-27]	0.14 [2.7e-31]	0.05 [1.9e-29]	0.03 [3.1e-02]
kalign	-0.15 [2.7e-30]	-0.01 [8.9e-02]	-0.18 [7.7e-31]	-0.09 [1.3e-25]		-0.14 [1.3e-30]	0.05 [9.7e-09]	-0.04 [2.6e-17]	-0.06 [4.3e-21]
fsa	-0.01 [6.2e-03]	0.13 [2.8e-31]	-0.04 [5.2e-06]	0.05 [7.6e-27]	0.14 [1.3e-30]		0.19 [2.7e-31]	0.10 [2.7e-31]	0.08 [4.0e-31]
clustalo	-0.20 [2.7e-31]	-0.06 [2.1e-18]	-0.23 [2.7e-31]	-0.14 [2.7e-31]	-0.05 [9.7e-09]	-0.19 [2.7e-31]		-0.09 [1.1e-26]	-0.11 [6.9e-20]
tcoffee	-0.11 [4.7e-31]	0.03 [4.2e-20]	-0.14 [2.8e-31]	-0.05 [1.9e-29]	0.04 [2.6e-17]	-0.10 [2.7e-31]	0.09 [1.1e-26]		-0.02 [8.1e-07]
probcons	-0.09 [2.7e-26]	0.05 [4.5e-14]	-0.12 [4.2e-24]	-0.03 [3.1e-02]	0.06 [4.3e-21]	-0.08 [4.0e-31]	0.11 [6.9e-20]	0.02 [8.1e-07]	

SPS Statistics for Charlie1 Sequence Divergence Analsysis
- Calculated on all replicate-parameter values

Tree Simulation: DNA Transposon Tree #2
Data File: paper-data/DNATransTree-2-Charlie1-R3S-eval/replicates.csv

Kruskal-Wallis H-test

	H =	117.2173154	p =	1.24E-21

Wilcoxon signed rank test: mean_diff [p-val]

	refiner	muscle	mafft	dialign	kalign	fsa	clustalo	tcoffee	probcons
refiner		0.20 [6.8e-31]	-0.03 [1.5e-04]	0.12 [3.3e-30]	0.20 [8.7e-30]	0.04 [7.0e-05]	0.28 [1.1e-30]	0.18 [7.1e-31]	0.16 [1.6e-28]
muscle	-0.20 [6.8e-31]		-0.22 [2.7e-31]	-0.07 [2.4e-26]	0.00 [5.3e-01]	-0.15 [1.8e-30]	0.08 [7.9e-18]	-0.01 [7.4e-05]	-0.03 [2.5e-05]
mafft	0.03 [1.5e-04]	0.22 [2.7e-31]		0.15 [5.8e-31]	0.23 [3.7e-30]	0.07 [4.2e-03]	0.30 [4.5e-31]	0.21 [2.7e-31]	0.19 [2.1e-17]
dialign	-0.12 [3.3e-30]	0.07 [2.4e-26]	-0.15 [5.8e-31]		0.08 [3.4e-13]	-0.08 [7.3e-15]	0.16 [2.8e-30]	0.06 [1.1e-17]	0.04 [8.0e-02]
kalign	-0.20 [8.7e-30]	-0.00 [5.3e-01]	-0.23 [3.7e-30]	-0.08 [3.4e-13]		-0.16 [3.0e-28]	0.08 [2.5e-13]	-0.02 [9.2e-06]	-0.04 [4.8e-05]
fsa	-0.04 [7.0e-05]	0.15 [1.8e-30]	-0.07 [4.2e-03]	0.08 [7.3e-15]	0.16 [3.0e-28]		0.23 [2.8e-28]	0.14 [4.0e-29]	0.12 [9.8e-31]
clustalo	-0.28 [1.1e-30]	-0.08 [7.9e-18]	-0.30 [4.5e-31]	-0.16 [2.8e-30]	-0.08 [2.5e-13]	-0.23 [2.8e-28]		-0.09 [3.1e-22]	-0.12 [4.3e-13]
tcoffee	-0.18 [7.1e-31]	0.01 [7.4e-05]	-0.21 [2.7e-31]	-0.06 [1.1e-17]	0.02 [9.2e-06]	-0.14 [4.0e-29]	0.09 [3.1e-22]		-0.02 [2.2e-03]
probcons	-0.16 [1.6e-28]	0.03 [2.5e-05]	-0.19 [2.1e-17]	-0.04 [8.0e-02]	0.04 [4.8e-05]	-0.12 [9.8e-31]	0.12 [4.3e-13]	0.02 [2.2e-03]	

S1.2 Derived Consensus Sequence Accuracy Metrics

The MSA-derived consensi were evaluated by globally aligning each to the consensus produced by the simulated alignment. Using the alignment of the simulated consensus to itself as the optimal baseline, the fraction of optimal alignment score lost (FOASL) was used to evaluate predicted MSA consensus reconstruction. For each MSA tool, 180 FOASL scores (10 replicates, 18 divergence bins) were collected for analysis. A Kruskal-Wallis H-test was performed to assess if there is a significant difference in the accuracy of reconstructions between MSA tools. Additionally, a Wilcoxon signed rank

S1.2.1 Statistics for Main Paper

Derived Consensus Statistics for Tigger1 Sequence Divergence Analysis

Tree Simulation: DNA Transposon Tree #1
Data File: paper-data/DNATransTree-1-Tigger1-R3S-eval/replicates.csv

Kruskal-Wallis H-test

	H =	268.3571074	p =	2.20E-53

Wilcoxon signed rank test: mean_diff [p-val]

	muscle	refiner	mafft	dialign	kalign	fsa	clustalo	tcoffee	probcons
muscle		0.25 [2.6e-20]	0.34 [9.5e-31]	0.15 [1.8e-20]	0.01 [9.8e-03]	-0.06 [5.2e-13]	0.08 [3.0e-13]	-0.31 [2.8e-31]	-0.08 [4.2e-20]
refiner	-0.25 [2.6e-20]		0.09 [1.4e-13]	-0.10 [1.6e-02]	-0.24 [2.1e-14]	-0.31 [6.5e-25]	-0.17 [1.6e-13]	-0.56 [2.7e-31]	-0.33 [1.1e-29]
mafft	-0.34 [9.5e-31]	-0.09 [1.4e-13]		-0.19 [8.8e-30]	-0.33 [6.3e-25]	-0.40 [2.7e-31]	-0.26 [1.6e-30]	-0.65 [2.7e-31]	-0.42 [7.7e-31]
dialign	-0.15 [1.8e-20]	0.10 [1.6e-02]	0.19 [8.8e-30]		-0.14 [2.4e-09]	-0.22 [2.7e-31]	-0.08 [1.3e-11]	-0.47 [4.0e-31]	-0.23 [5.7e-25]
kalign	-0.01 [9.8e-03]	0.24 [2.1e-14]	0.33 [6.3e-25]	0.14 [2.4e-09]		-0.07 [1.5e-08]	0.06 [2.2e-02]	-0.33 [2.7e-31]	-0.09 [3.6e-13]
fsa	0.06 [5.2e-13]	0.31 [6.5e-25]	0.40 [2.7e-31]	0.22 [2.7e-31]	0.07 [1.5e-08]		0.14 [1.5e-24]	-0.25 [1.5e-24]	-0.02 [2.8e-03]
clustalo	-0.08 [3.0e-13]	0.17 [1.6e-13]	0.26 [1.6e-30]	0.08 [1.3e-11]	-0.06 [2.2e-02]	-0.14 [1.5e-24]		-0.39 [2.7e-31]	-0.16 [2.4e-28]
tcoffee	0.31 [2.8e-31]	0.56 [2.7e-31]	0.65 [2.7e-31]	0.47 [4.0e-31]	0.33 [2.7e-31]	0.25 [1.5e-24]	0.39 [2.7e-31]		0.23 [9.9e-31]
probcons	0.08 [4.2e-20]	0.33 [1.1e-29]	0.42 [7.7e-31]	0.23 [5.7e-25]	0.09 [3.6e-13]	0.02 [2.8e-03]	0.16 [2.4e-28]	-0.23 [9.9e-31]	

Derived Consensus Statistics for L2 Sequence Divergence Analysis

Tree Simulation: LINE Tree #1
Data File: paper-data/LINETree-1-L2-R3S-eval/replicates.csv

Kruskal-Wallis H-test

H = 350.7136137 p = 6.38E-71

Wilcoxon signed rank test: mean_diff [p-val]

	muscle	refiner	mafft	dialign	kalign	fsa	clustalo	tcoffee	probcons
muscle		0.19 [1.1e-20]	0.26 [5.7e-31]	0.10 [1.2e-17]	-0.06 [2.6e-03]	-0.13 [3.7e-28]	0.04 [8.2e-08]	-0.39 [2.7e-31]	-0.16 [3.5e-30]
refiner	-0.19 [1.1e-20]		0.07 [1.8e-16]	-0.09 [9.8e-01]	-0.25 [1.4e-15]	-0.32 [4.0e-27]	-0.14 [7.3e-15]	-0.58 [2.7e-31]	-0.35 [1.2e-30]
mafft	-0.26 [5.7e-31]	-0.07 [1.8e-16]		-0.16 [1.4e-29]	-0.32 [2.4e-29]	-0.39 [5.9e-31]	-0.22 [2.7e-31]	-0.65 [2.7e-31]	-0.42 [2.7e-31]
dialign	-0.10 [1.2e-17]	0.09 [9.8e-01]	0.16 [1.4e-29]		-0.16 [1.0e-13]	-0.23 [2.8e-31]	-0.05 [1.1e-12]	-0.49 [2.7e-31]	-0.26 [3.7e-27]
kalign	0.06 [2.6e-03]	0.25 [1.4e-15]	0.32 [2.4e-29]	0.16 [1.0e-13]		-0.07 [1.1e-07]	0.11 [4.5e-07]	-0.32 [2.7e-31]	-0.10 [7.7e-16]
fsa	0.13 [3.7e-28]	0.32 [4.0e-27]	0.39 [5.9e-31]	0.23 [2.8e-31]	0.07 [1.1e-07]		0.18 [5.5e-28]	-0.26 [1.6e-27]	-0.03 [3.1e-05]
clustalo	-0.04 [8.2e-08]	0.14 [7.3e-15]	0.22 [2.7e-31]	0.05 [1.1e-12]	-0.11 [4.5e-07]	-0.18 [5.5e-28]		-0.43 [2.7e-31]	-0.21 [5.9e-30]
tcoffee	0.39 [2.7e-31]	0.58 [2.7e-31]	0.65 [2.7e-31]	0.49 [2.7e-31]	0.32 [2.7e-31]	0.26 [1.6e-27]	0.43 [2.7e-31]		0.23 [5.2e-31]
probcons	0.16 [3.5e-30]	0.35 [1.2e-30]	0.42 [2.7e-31]	0.26 [3.7e-27]	0.10 [7.7e-16]	0.03 [3.1e-05]	0.21 [5.9e-30]	-0.23 [5.2e-31]	

Derived Consensus Statistics for Tigger1 Sequence Fragmentation Analysis

Tree Simulation: DNA Transposon Tree #1
Fragmentation Simulation: Low Divergence Sequences [gput100]
Data File: paper-data/DNATransTree-1-Tigger1-R3S-gput100-mfl2-eval/replicates.csv

Kruskal-Wallis H-test

H = 724.1474921 p = 4.52E-151

Wilcoxon signed rank test: mean_diff [p-val]

	muscle	refiner	mafft	dialign	kalign	fsa	clustalo	tcoffee	probcons
muscle		0.26 [8.9e-20]	0.02 [5.6e-02]	0.26 [7.8e-21]	0.14 [4.4e-08]	-0.35 [3.7e-10]	0.25 [2.8e-15]	-1.03 [2.0e-21]	-0.52 [2.7e-20]
refiner	-0.26 [8.9e-20]		-0.24 [2.5e-14]	0.00 [4.4e-01]	-0.12 [1.4e-13]	-0.61 [8.0e-21]	-0.01 [3.8e-20]	-1.29 [2.0e-21]	-0.78 [2.0e-21]
mafft	-0.02 [5.6e-02]	0.24 [2.5e-14]		0.24 [3.7e-14]	0.12 [2.3e-04]	-0.37 [4.4e-19]	0.22 [1.3e-08]	-1.05 [2.0e-21]	-0.54 [2.0e-21]
dialign	-0.26 [7.8e-21]	-0.00 [4.4e-01]	-0.24 [3.7e-14]		-0.12 [4.5e-14]	-0.61 [3.0e-21]	-0.02 [4.7e-19]	-1.29 [2.0e-21]	-0.78 [2.0e-21]
kalign	-0.14 [4.4e-08]	0.12 [1.4e-13]	-0.12 [2.3e-04]	0.12 [4.5e-14]		-0.49 [4.7e-20]	0.10 [8.7e-03]	-1.18 [2.0e-21]	-0.66 [2.0e-21]
fsa	0.35 [3.7e-10]	0.61 [8.0e-21]	0.37 [4.4e-19]	0.61 [3.0e-21]	0.49 [4.7e-20]		0.59 [3.0e-16]	-0.68 [2.0e-21]	-0.17 [1.9e-13]
clustalo	-0.25 [2.8e-15]	0.01 [3.8e-20]	-0.22 [1.3e-08]	0.02 [4.7e-19]	-0.10 [8.7e-03]	-0.59 [3.0e-16]		-1.28 [2.0e-21]	-0.76 [2.0e-21]
tcoffee	1.03 [2.0e-21]	1.29 [2.0e-21]	1.05 [2.0e-21]	1.29 [2.0e-21]	1.18 [2.0e-21]	0.68 [2.0e-21]	1.28 [2.0e-21]		0.51 [2.0e-21]
probcons	0.52 [2.7e-20]	0.78 [2.0e-21]	0.54 [2.0e-21]	0.78 [2.0e-21]	0.66 [2.0e-21]	0.17 [1.9e-13]	0.76 [2.0e-21]	-0.51 [2.0e-21]	

Derived Consensus Statistics for Tigger1 Sequence Fragmentation Analysis

Tree Simulation: DNA Transposon Tree #1
Fragmentation Simulation: High Divergence Sequences [gput3000]
Data File: paper-data/DNATransTree-1-Tigger1-R3S-gput3000-mfl2-eval/replicates.csv

Kruskal-Wallis H-test

H = 661.1501866 p = 1.65E-137

Wilcoxon signed rank test: mean_diff [p-val]

	muscle	refiner	mafft	dialign	kalign	fsa	clustalo	tcoffee	probcons
muscle		0.68 [2.0e-21]	0.26 [5.6e-06]	0.07 [2.0e-01]	-0.12 [4.2e-18]	-0.18 [1.4e-18]	-0.08 [5.0e-09]	-0.24 [2.0e-21]	-0.02 [4.2e-01]
refiner	-0.68 [2.0e-21]		-0.42 [1.4e-12]	-0.60 [2.0e-18]	-0.80 [2.0e-21]	-0.86 [2.0e-21]	-0.76 [2.0e-21]	-0.91 [2.0e-21]	-0.70 [2.0e-21]
mafft	-0.26 [5.6e-06]	0.42 [1.4e-12]		-0.18 [3.7e-18]	-0.38 [8.0e-21]	-0.44 [2.0e-21]	-0.34 [4.7e-20]	-0.49 [2.0e-21]	-0.28 [5.9e-04]
dialign	-0.07 [2.0e-01]	0.60 [2.0e-18]	0.18 [3.7e-18]		-0.20 [7.6e-05]	-0.26 [2.0e-21]	-0.16 [8.5e-04]	-0.31 [2.0e-21]	-0.09 [3.6e-01]
kalign	0.12 [4.2e-18]	0.80 [2.0e-21]	0.38 [8.0e-21]	0.20 [7.6e-05]		-0.06 [9.6e-12]	0.04 [2.2e-09]	-0.11 [2.0e-21]	0.10 [1.2e-09]
fsa	0.18 [1.4e-18]	0.86 [2.0e-21]	0.44 [2.0e-21]	0.26 [2.0e-21]	0.06 [9.6e-12]		0.10 [1.4e-16]	-0.05 [5.6e-04]	0.16 [1.0e-09]
clustalo	0.08 [5.0e-09]	0.76 [2.0e-21]	0.34 [4.7e-20]	0.16 [8.5e-04]	-0.04 [2.2e-09]	-0.10 [1.4e-16]		-0.15 [2.0e-21]	0.06 [2.5e-04]
tcoffee	0.24 [2.0e-21]	0.91 [2.0e-21]	0.49 [2.0e-21]	0.31 [2.0e-21]	0.11 [2.0e-21]	0.05 [5.6e-04]	0.15 [2.0e-21]		0.21 [2.0e-21]
probcons	0.02 [4.2e-01]	0.70 [2.0e-21]	0.28 [5.9e-04]	0.09 [3.6e-01]	-0.10 [1.2e-09]	-0.16 [1.0e-09]	-0.06 [2.5e-04]	-0.21 [2.0e-21]	

S1.2.2 Statistics for Additional Trees and Seed Sequences

Derived Consensus Statistics for Charlie1 Sequence Divergence Analysis

Tree Simulation: DNA Transposon Tree #1
Data File: paper-data/DNATransTree-1-Charlie1-R3S-eval/replicates.csv

Kruskal-Wallis H-test

H = 280.4546806 p = 5.91E-56

Wilcoxon signed rank test: mean_diff [p-val]

	muscle	refiner	mafft	dialign	kalign	fsa	clustalo	tcoffee	probcons
muscle		0.29 [1.1e-22]	0.36 [1.4e-30]	0.16 [1.2e-19]	0.03 [3.1e-04]	-0.07 [3.0e-16]	0.08 [1.0e-12]	-0.31 [5.5e-31]	-0.10 [1.5e-23]
refiner	-0.29 [1.1e-22]		0.07 [1.2e-11]	-0.13 [3.2e-05]	-0.26 [1.1e-16]	-0.36 [2.0e-27]	-0.21 [6.6e-18]	-0.60 [2.7e-31]	-0.39 [8.1e-31]
mafft	-0.36 [1.4e-30]	-0.07 [1.2e-11]		-0.20 [3.1e-30]	-0.33 [1.4e-24]	-0.43 [2.7e-31]	-0.28 [5.2e-31]	-0.67 [2.7e-31]	-0.46 [2.9e-31]
dialign	-0.16 [1.2e-19]	0.13 [3.2e-05]	0.20 [3.1e-30]		-0.14 [1.9e-08]	-0.23 [2.7e-31]	-0.08 [2.1e-11]	-0.47 [3.5e-31]	-0.26 [4.5e-26]
kalign	-0.03 [3.1e-04]	0.26 [1.1e-16]	0.33 [1.4e-24]	0.14 [1.9e-08]		-0.09 [1.5e-10]	0.06 [1.1e-01]	-0.34 [2.7e-31]	-0.13 [8.8e-19]
fsa	0.07 [3.0e-16]	0.36 [2.0e-27]	0.43 [2.7e-31]	0.23 [2.7e-31]	0.09 [1.5e-10]		0.15 [7.8e-26]	-0.25 [6.3e-24]	-0.03 [7.0e-05]
clustalo	-0.08 [1.0e-12]	0.21 [6.6e-18]	0.28 [5.2e-31]	0.08 [2.1e-11]	-0.06 [1.1e-01]	-0.15 [7.8e-26]		-0.40 [2.7e-31]	-0.18 [2.0e-28]
tcoffee	0.31 [5.5e-31]	0.60 [2.7e-31]	0.67 [2.7e-31]	0.47 [3.5e-31]	0.34 [2.7e-31]	0.25 [6.3e-24]	0.40 [2.7e-31]		0.21 [3.2e-30]
probcons	0.10 [1.5e-23]	0.39 [8.1e-31]	0.46 [2.9e-31]	0.26 [4.5e-26]	0.13 [8.8e-19]	0.03 [7.0e-05]	0.18 [2.0e-28]	-0.21 [3.2e-30]	

Derived Consensus Statistics for CR1 Sequence Divergence Analysis

Tree Simulation: LINE Tree #1
Data File: paper-data/LINETree-1-CR1-R3S-eval/replicates.csv

Kruskal-Wallis H-test

	H =	340.6250421	p =	9.07E-69

Wilcoxon signed rank test: mean_diff [p-val]

	muscle	refiner	mafft	dialign	kalign	fsa	clustalo	tcoffee	probcons
muscle		0.18 [2.2e-15]	0.25 [8.2e-31]	0.11 [2.8e-22]	-0.03 [9.0e-01]	-0.10 [2.2e-25]	0.06 [5.5e-11]	-0.37 [2.7e-31]	-0.10 [5.3e-24]
refiner	-0.18 [2.2e-15]		0.08 [1.2e-17]	-0.07 [5.1e-01]	-0.20 [6.7e-08]	-0.28 [8.2e-24]	-0.12 [3.6e-08]	-0.54 [2.7e-31]	-0.27 [2.3e-29]
mafft	-0.25 [8.2e-31]	-0.08 [1.2e-17]		-0.14 [2.1e-29]	-0.28 [1.6e-29]	-0.35 [4.2e-31]	-0.20 [2.7e-31]	-0.62 [2.7e-31]	-0.35 [2.7e-31]
dialign	-0.11 [2.8e-22]	0.07 [5.1e-01]	0.14 [2.1e-29]		-0.13 [2.6e-09]	-0.21 [2.8e-31]	-0.05 [3.2e-15]	-0.48 [3.0e-31]	-0.20 [1.2e-26]
kalign	0.03 [9.0e-01]	0.20 [6.7e-08]	0.28 [1.6e-29]	0.13 [2.6e-09]		-0.08 [8.4e-12]	0.08 [5.1e-03]	-0.34 [2.8e-31]	-0.07 [4.4e-10]
fsa	0.10 [2.2e-25]	0.28 [8.2e-24]	0.35 [4.2e-31]	0.21 [2.8e-31]	0.08 [8.4e-12]		0.16 [1.1e-25]	-0.27 [5.9e-28]	0.00 [3.2e-01]
clustalo	-0.06 [5.5e-11]	0.12 [3.6e-08]	0.20 [2.7e-31]	0.05 [3.2e-15]	-0.08 [5.1e-03]	-0.16 [1.1e-25]		-0.43 [2.7e-31]	-0.15 [3.6e-29]
tcoffee	0.37 [2.7e-31]	0.54 [2.7e-31]	0.62 [2.7e-31]	0.48 [3.0e-31]	0.34 [2.8e-31]	0.27 [5.9e-28]	0.43 [2.7e-31]		0.27 [2.9e-31]
probcons	0.10 [5.3e-24]	0.27 [2.3e-29]	0.35 [2.7e-31]	0.20 [1.2e-26]	0.07 [4.4e-10]	-0.00 [3.2e-01]	0.15 [3.6e-29]	-0.27 [2.9e-31]	

Derived Consensus Statistics for L2 Sequence Fragmentation Analysis

Tree Simulation: LINE Tree #1
Fragmentation Simulation: Low Divergence Sequences [gput100]
Data File: paper-data/LINETree-1-L2-R3S-gput100-mfl2-eval/replicates.csv

Kruskal-Wallis H-test

	H =	758.7946438	p =	1.56E-158

Wilcoxon signed rank test: mean_diff [p-val]

	muscle	refiner	mafft	dialign	kalign	fsa	clustalo	tcoffee	probcons
muscle		0.42 [2.0e-21]	0.11 [1.8e-03]	0.42 [2.0e-21]	-0.02 [9.2e-01]	-0.27 [2.3e-07]	0.40 [4.2e-21]	-0.95 [2.0e-21]	-0.41 [4.0e-19]
refiner	-0.42 [2.0e-21]		-0.30 [1.4e-15]	0.00 [8.4e-02]	-0.44 [3.6e-19]	-0.68 [4.3e-21]	-0.01 [1.9e-17]	-1.36 [2.0e-21]	-0.82 [2.0e-21]
mafft	-0.11 [1.8e-03]	0.30 [1.4e-15]		0.31 [1.6e-15]	-0.13 [1.4e-04]	-0.38 [1.1e-18]	0.29 [1.4e-12]	-1.06 [2.0e-21]	-0.52 [2.8e-21]
dialign	-0.42 [2.0e-21]	-0.00 [8.4e-02]	-0.31 [1.6e-15]		-0.44 [2.3e-19]	-0.68 [3.1e-21]	-0.01 [3.6e-21]	-1.36 [2.0e-21]	-0.82 [2.0e-21]
kalign	0.02 [9.2e-01]	0.44 [3.6e-19]	0.13 [1.4e-04]	0.44 [2.3e-19]		-0.25 [2.4e-09]	0.43 [3.0e-15]	-0.92 [2.0e-21]	-0.39 [2.3e-16]
fsa	0.27 [2.3e-07]	0.68 [4.3e-21]	0.38 [1.1e-18]	0.68 [3.1e-21]	0.25 [2.4e-09]		0.67 [3.3e-18]	-0.68 [2.0e-21]	-0.14 [3.0e-04]
clustalo	-0.40 [4.2e-21]	0.01 [1.9e-17]	-0.29 [1.4e-12]	0.01 [3.6e-21]	-0.43 [3.0e-15]	-0.67 [3.3e-18]		-1.35 [2.0e-21]	-0.81 [2.0e-21]
tcoffee	0.95 [2.0e-21]	1.36 [2.0e-21]	1.06 [2.0e-21]	1.36 [2.0e-21]	0.92 [2.0e-21]	0.68 [2.0e-21]	1.35 [2.0e-21]		0.54 [2.0e-21]
probcons	0.41 [4.0e-19]	0.82 [2.0e-21]	0.52 [2.8e-21]	0.82 [2.0e-21]	0.39 [2.3e-16]	0.14 [3.0e-04]	0.81 [2.0e-21]	-0.54 [2.0e-21]	

Derived Consensus Statistics for L2 Sequence Fragmentation Analysis

Tree Simulation: LINE Tree #1
Fragmentation Simulation: Medium Divergence Sequences [gput1500]
Data File: paper-data/LINETree-1-L2-R3S-gput1500-mfl2-eval/replicates.csv

Kruskal-Wallis H-test

	H =	701.0448939	p =	4.26E-146

Wilcoxon signed rank test: mean_diff [p-val]

	muscle	refiner	mafft	dialign	kalign	fsa	clustalo	tcoffee	probcons
muscle		0.74 [8.6e-20]	0.48 [3.0e-19]	0.61 [3.8e-21]	-0.14 [2.3e-08]	-0.11 [7.4e-05]	0.68 [2.0e-21]	-0.42 [2.0e-21]	-0.03 [8.5e-01]
refiner	-0.74 [8.6e-20]		-0.27 [3.1e-06]	-0.13 [7.2e-03]	-0.88 [4.5e-19]	-0.85 [2.8e-18]	-0.06 [9.6e-02]	-1.16 [2.0e-21]	-0.77 [5.5e-21]
mafft	-0.48 [3.0e-19]	0.27 [3.1e-06]		0.14 [4.5e-04]	-0.61 [2.0e-21]	-0.59 [2.0e-21]	0.21 [2.3e-06]	-0.90 [2.0e-21]	-0.51 [1.8e-19]
dialign	-0.61 [3.8e-21]	0.13 [7.2e-03]	-0.14 [4.5e-04]		-0.75 [2.2e-21]	-0.73 [2.0e-21]	0.07 [2.6e-02]	-1.03 [2.0e-21]	-0.65 [3.9e-21]
kalign	0.14 [2.3e-08]	0.88 [4.5e-19]	0.61 [2.0e-21]	0.75 [2.2e-21]		0.02 [7.2e-01]	0.82 [2.1e-21]	-0.28 [2.0e-21]	0.10 [8.9e-06]
fsa	0.11 [7.4e-05]	0.85 [2.8e-18]	0.59 [2.0e-21]	0.73 [2.0e-21]	-0.02 [7.2e-01]		0.79 [2.1e-21]	-0.31 [1.7e-19]	0.08 [2.9e-03]
clustalo	-0.68 [2.0e-21]	0.06 [9.6e-02]	-0.21 [2.3e-06]	-0.07 [2.6e-02]	-0.82 [2.1e-21]	-0.79 [2.1e-21]		-1.10 [2.0e-21]	-0.71 [2.1e-21]
tcoffee	0.42 [2.0e-21]	1.16 [2.0e-21]	0.90 [2.0e-21]	1.03 [2.0e-21]	0.28 [2.0e-21]	0.31 [1.7e-19]	1.10 [2.0e-21]		0.39 [2.0e-21]
probcons	0.03 [8.5e-01]	0.77 [5.5e-21]	0.51 [1.8e-19]	0.65 [3.9e-21]	-0.10 [8.9e-06]	-0.08 [2.9e-03]	0.71 [2.1e-21]	-0.39 [2.0e-21]	

Derived Consensus Statistics for L2 Sequence Fragmentation Analysis

Tree Simulation: LINE Tree #1
Fragmentation Simulation: High Divergence Sequences [gput3000]
Data File: paper-data/LINETree-1-L2-R3S-gput3000-mfl2-eval/replicates.csv

Kruskal-Wallis H-test

	H =	642.5496625	p =	1.65E-133

Wilcoxon signed rank test: mean_diff [p-val]

	muscle	refiner	mafft	dialign	kalign	fsa	clustalo	tcoffee	probcons
muscle		0.42 [2.7e-21]	0.23 [2.2e-04]	0.09 [1.1e-01]	-0.14 [2.7e-19]	-0.20 [1.2e-18]	-0.06 [2.1e-05]	-0.25 [2.0e-21]	0.02 [1.3e-02]
refiner	-0.42 [2.7e-21]		-0.19 [5.6e-05]	-0.33 [2.4e-11]	-0.56 [2.0e-21]	-0.62 [2.0e-21]	-0.48 [2.4e-21]	-0.67 [2.0e-21]	-0.40 [4.0e-21]
mafft	-0.23 [2.2e-04]	0.19 [5.6e-05]		-0.13 [2.7e-16]	-0.37 [2.4e-21]	-0.43 [2.0e-21]	-0.29 [1.1e-18]	-0.48 [2.0e-21]	-0.21 [3.3e-02]
dialign	-0.09 [1.1e-01]	0.33 [2.4e-11]	0.13 [2.7e-16]		-0.23 [3.9e-06]	-0.29 [2.0e-21]	-0.15 [1.4e-03]	-0.34 [2.0e-21]	-0.07 [5.8e-01]
kalign	0.14 [2.7e-19]	0.56 [2.0e-21]	0.37 [2.4e-21]	0.23 [3.9e-06]		-0.06 [3.6e-11]	0.08 [1.1e-15]	-0.11 [2.0e-21]	0.16 [9.8e-15]
fsa	0.20 [1.2e-18]	0.62 [2.0e-21]	0.43 [2.0e-21]	0.29 [2.0e-21]	0.06 [3.6e-11]		0.14 [2.3e-19]	-0.05 [2.4e-02]	0.22 [8.1e-13]
clustalo	0.06 [2.1e-05]	0.48 [2.4e-21]	0.29 [1.1e-18]	0.15 [1.4e-03]	-0.08 [1.1e-15]	-0.14 [2.3e-19]		-0.19 [2.0e-21]	0.08 [2.4e-04]
tcoffee	0.25 [2.0e-21]	0.67 [2.0e-21]	0.48 [2.0e-21]	0.34 [2.0e-21]	0.11 [2.0e-21]	0.05 [2.4e-02]	0.19 [2.0e-21]		0.27 [2.0e-21]
probcons	-0.02 [1.3e-02]	0.40 [4.0e-21]	0.21 [3.3e-02]	0.07 [5.8e-01]	-0.16 [9.8e-15]	-0.22 [8.1e-13]	-0.08 [2.4e-04]	-0.27 [2.0e-21]	

Derived Consensus Statistics for Tigger1 Sequence Fragmentation Analysis

Tree Simulation: DNA Transposon Tree #1
Fragmentation Simulation: Medium Divergence Sequences [gput1500]
Data File: paper-data/DNATransTree-1-Tigger1-R3S-gput1500-mfl2-eval/replicates.csv

Kruskal-Wallis H-test

H = 668.6148292 p = 4.08E-139

Wilcoxon signed rank test: mean_diff [p-val]

	muscle	refiner	mafft	dialign	kalign	fsa	clustalo	tcoffee	probcons
muscle		0.81 [2.1e-20]	0.52 [1.9e-20]	0.51 [1.8e-20]	-0.11 [1.6e-06]	-0.13 [3.9e-06]	0.62 [2.4e-21]	-0.43 [2.0e-21]	-0.08 [6.0e-07]
refiner	-0.81 [2.1e-20]		-0.29 [3.2e-08]	-0.30 [1.6e-11]	-0.92 [1.0e-19]	-0.94 [1.8e-20]	-0.19 [5.4e-11]	-1.24 [2.0e-21]	-0.89 [3.7e-21]
mafft	-0.52 [1.9e-20]	0.29 [3.2e-08]		-0.01 [8.8e-02]	-0.63 [3.3e-21]	-0.65 [2.0e-21]	0.10 [3.4e-02]	-0.95 [2.0e-21]	-0.60 [3.2e-21]
dialign	-0.51 [1.8e-20]	0.30 [1.6e-11]	0.01 [8.8e-02]		-0.62 [3.3e-21]	-0.64 [2.0e-21]	0.11 [2.5e-03]	-0.94 [2.0e-21]	-0.59 [3.7e-21]
kalign	0.11 [1.6e-06]	0.92 [1.0e-19]	0.63 [3.3e-21]	0.62 [3.3e-21]		-0.02 [1.1e-01]	0.73 [5.4e-21]	-0.33 [2.0e-21]	0.02 [1.2e-01]
fsa	0.13 [3.9e-06]	0.94 [1.8e-20]	0.65 [2.0e-21]	0.64 [2.0e-21]	0.02 [1.1e-01]		0.75 [2.0e-21]	-0.30 [1.3e-20]	0.05 [7.8e-02]
clustalo	-0.62 [2.4e-21]	0.19 [5.4e-11]	-0.10 [3.4e-02]	-0.11 [2.5e-03]	-0.73 [5.4e-21]	-0.75 [2.0e-21]		-1.05 [2.0e-21]	-0.70 [2.0e-21]
tcoffee	0.43 [2.0e-21]	1.24 [2.0e-21]	0.95 [2.0e-21]	0.94 [2.0e-21]	0.33 [2.0e-21]	0.30 [1.3e-20]	1.05 [2.0e-21]		0.35 [2.0e-21]
probcons	0.08 [6.0e-07]	0.89 [3.7e-21]	0.60 [3.2e-21]	0.59 [3.7e-21]	-0.02 [1.2e-01]	-0.05 [7.8e-02]	0.70 [2.0e-21]	-0.35 [2.0e-21]	

Derived Consensus Statistics for Tigger1 Sequence Divergence Analysis

Tree Simulation: DNA Transposon Tree #2
Data File: paper-data/DNATransTree-2-Tigger1-R3S-eval/replicates.csv

Kruskal-Wallis H-test

H = 261.0485343 p = 7.82E-52

Wilcoxon signed rank test: mean_diff [p-val]

	muscle	refiner	mafft	dialign	kalign	fsa	clustalo	tcoffee	probcons
muscle		0.24 [1.9e-20]	0.34 [1.9e-30]	0.15 [6.3e-20]	0.01 [1.1e-02]	-0.06 [4.9e-11]	0.08 [4.2e-12]	-0.31 [3.0e-31]	-0.08 [3.6e-19]
refiner	-0.24 [1.9e-20]		0.10 [5.3e-15]	-0.09 [6.1e-03]	-0.23 [5.3e-14]	-0.30 [8.6e-25]	-0.17 [4.7e-13]	-0.55 [2.7e-31]	-0.32 [1.5e-29]
mafft	-0.34 [1.9e-30]	-0.10 [5.3e-15]		-0.19 [7.5e-30]	-0.33 [5.1e-25]	-0.40 [2.7e-31]	-0.27 [4.0e-31]	-0.65 [2.7e-31]	-0.42 [2.8e-31]
dialign	-0.15 [6.3e-20]	0.09 [6.1e-03]	0.19 [7.5e-30]		-0.14 [2.3e-09]	-0.21 [4.0e-31]	-0.08 [5.6e-10]	-0.46 [5.1e-31]	-0.23 [6.7e-25]
kalign	-0.01 [1.1e-02]	0.23 [5.3e-14]	0.33 [5.1e-25]	0.14 [2.3e-09]		-0.07 [3.4e-08]	0.06 [2.9e-02]	-0.32 [2.7e-31]	-0.09 [1.6e-12]
fsa	0.06 [4.9e-11]	0.30 [8.6e-25]	0.40 [2.7e-31]	0.21 [4.0e-31]	0.07 [3.4e-08]		0.14 [4.7e-26]	-0.25 [5.7e-24]	-0.02 [6.3e-03]
clustalo	-0.08 [4.2e-12]	0.17 [4.7e-13]	0.27 [4.0e-31]	0.08 [5.6e-10]	-0.06 [2.9e-02]	-0.14 [4.7e-26]		-0.38 [2.7e-31]	-0.15 [9.3e-28]
tcoffee	0.31 [3.0e-31]	0.55 [2.7e-31]	0.65 [2.7e-31]	0.46 [5.1e-31]	0.32 [2.7e-31]	0.25 [5.7e-24]	0.38 [2.7e-31]		0.23 [1.9e-30]
probcons	0.08 [3.6e-19]	0.32 [1.5e-29]	0.42 [2.8e-31]	0.23 [6.7e-25]	0.09 [1.6e-12]	0.02 [6.3e-03]	0.15 [9.3e-28]	-0.23 [1.9e-30]	

Derived Consensus Statistics for CR1 Sequence Divergence Analysis

Tree Simulation: LINE Tree #2
Data File: paper-data/LINETree-2-CR1-R3S-eval/replicates.csv

Kruskal-Wallis H-test

	H =	474.0447306	p =	2.60E-97

Wilcoxon signed rank test: mean_diff [p-val]

	muscle	refiner	mafft	dialign	kalign	fsa	clustalo	tcoffee	probcons
muscle		0.05 [2.4e-07]	0.14 [3.0e-30]	0.06 [1.1e-19]	-0.03 [9.6e-01]	-0.16 [3.1e-29]	0.02 [1.3e-01]	-0.43 [2.7e-31]	-0.14 [2.6e-29]
refiner	-0.05 [2.4e-07]		0.09 [1.4e-27]	0.01 [1.1e-06]	-0.09 [8.4e-04]	-0.21 [4.0e-23]	-0.03 [2.8e-07]	-0.48 [2.7e-31]	-0.19 [7.2e-29]
mafft	-0.14 [3.0e-30]	-0.09 [1.4e-27]		-0.08 [1.2e-29]	-0.17 [4.7e-29]	-0.29 [4.3e-31]	-0.12 [2.7e-31]	-0.57 [2.7e-31]	-0.27 [2.7e-31]
dialign	-0.06 [1.1e-19]	-0.01 [1.1e-06]	0.08 [1.2e-29]		-0.09 [2.9e-17]	-0.22 [4.1e-31]	-0.04 [7.6e-18]	-0.49 [2.7e-31]	-0.20 [2.7e-31]
kalign	0.03 [9.6e-01]	0.09 [8.4e-04]	0.17 [4.7e-29]	0.09 [2.9e-17]		-0.12 [6.2e-30]	0.05 [6.4e-03]	-0.40 [2.7e-31]	-0.10 [1.0e-24]
fsa	0.16 [3.1e-29]	0.21 [4.0e-23]	0.29 [4.3e-31]	0.22 [4.1e-31]	0.12 [6.2e-30]		0.17 [2.8e-24]	-0.28 [9.2e-30]	0.02 [5.7e-01]
clustalo	-0.02 [1.3e-01]	0.03 [2.8e-07]	0.12 [2.7e-31]	0.04 [7.6e-18]	-0.05 [6.4e-03]	-0.17 [2.8e-24]		-0.45 [2.7e-31]	-0.15 [6.2e-30]
tcoffee	0.43 [2.7e-31]	0.48 [2.7e-31]	0.57 [2.7e-31]	0.49 [2.7e-31]	0.40 [2.7e-31]	0.28 [9.2e-30]	0.45 [2.7e-31]		0.30 [2.7e-31]
probcons	0.14 [2.6e-29]	0.19 [7.2e-29]	0.27 [2.7e-31]	0.20 [2.7e-31]	0.10 [1.0e-24]	-0.02 [5.7e-01]	0.15 [6.2e-30]	-0.30 [2.7e-31]	

Derived Consensus Statistics for L2 Sequence Divergence Analysis

Tree Simulation: LINE Tree #2
Data File: paper-data/LINETree-2-L2-R3S-eval/replicates.csv

Kruskal-Wallis H-test

	H =	456.7252135	p =	1.34E-93

Wilcoxon signed rank test: mean_diff [p-val]

	muscle	refiner	mafft	dialign	kalign	fsa	clustalo	tcoffee	probcons
muscle		0.05 [1.9e-09]	0.13 [3.8e-30]	0.04 [1.4e-11]	-0.10 [4.3e-05]	-0.19 [4.1e-29]	-0.01 [6.8e-01]	-0.46 [2.7e-31]	-0.19 [2.8e-31]
refiner	-0.05 [1.9e-09]		0.09 [2.3e-26]	-0.01 [1.2e-03]	-0.15 [1.0e-09]	-0.24 [8.4e-26]	-0.05 [3.4e-11]	-0.50 [2.7e-31]	-0.24 [8.1e-31]
mafft	-0.13 [3.8e-30]	-0.09 [2.3e-26]		-0.10 [4.3e-28]	-0.23 [8.2e-29]	-0.32 [4.9e-31]	-0.14 [2.8e-31]	-0.59 [2.7e-31]	-0.33 [2.7e-31]
dialign	-0.04 [1.4e-11]	0.01 [1.2e-03]	0.10 [4.3e-28]		-0.13 [2.1e-24]	-0.22 [2.8e-31]	-0.04 [6.5e-17]	-0.49 [2.7e-31]	-0.23 [2.7e-31]
kalign	0.10 [4.3e-05]	0.15 [1.0e-09]	0.23 [8.2e-29]	0.13 [2.1e-24]		-0.09 [1.4e-21]	0.09 [6.4e-07]	-0.36 [2.7e-31]	-0.09 [6.2e-19]
fsa	0.19 [4.1e-29]	0.24 [8.4e-26]	0.32 [4.9e-31]	0.22 [2.8e-31]	0.09 [1.4e-21]		0.18 [9.4e-25]	-0.27 [1.0e-29]	-0.00 [2.3e-02]
clustalo	0.01 [6.8e-01]	0.05 [3.4e-11]	0.14 [2.8e-31]	0.04 [6.5e-17]	-0.09 [6.4e-07]	-0.18 [9.4e-25]		-0.45 [2.7e-31]	-0.19 [3.6e-31]
tcoffee	0.46 [2.7e-31]	0.50 [2.7e-31]	0.59 [2.7e-31]	0.49 [2.7e-31]	0.36 [2.7e-31]	0.27 [1.0e-29]	0.45 [2.7e-31]		0.26 [2.8e-31]
probcons	0.19 [2.8e-31]	0.24 [8.1e-31]	0.33 [2.7e-31]	0.23 [2.7e-31]	0.09 [6.2e-19]	0.00 [2.3e-02]	0.19 [3.6e-31]	-0.26 [2.8e-31]	

Derived Consensus Statistics for Charlie1 Sequence Divergence Analysis

Tree Simulation: DNA Transposon Tree #2
Data File: paper-data/DNATransTree-2-Charlie1-R3S-eval/replicates.csv

Kruskal-Wallis H-test

	H =	276.6653794	p =	3.78E-55

Wilcoxon signed rank test: mean_diff [p-val]

	muscle	refiner	mafft	dialign	kalign	fsa	clustalo	tcoffee	probcons
muscle		0.27 [2.4e-20]	0.35 [1.7e-30]	0.15 [4.4e-18]	0.01 [5.8e-02]	-0.08 [6.8e-19]	0.08 [7.6e-13]	-0.33 [2.7e-31]	-0.11 [1.2e-26]
refiner	-0.27 [2.4e-20]		0.07 [4.2e-11]	-0.13 [8.1e-05]	-0.27 [3.5e-16]	-0.36 [7.1e-27]	-0.20 [7.1e-16]	-0.61 [2.7e-31]	-0.39 [1.7e-30]
mafft	-0.35 [1.7e-30]	-0.07 [4.2e-11]		-0.20 [2.1e-30]	-0.34 [2.2e-23]	-0.43 [2.7e-31]	-0.27 [8.9e-31]	-0.68 [2.7e-31]	-0.46 [8.1e-31]
dialign	-0.15 [4.4e-18]	0.13 [8.1e-05]	0.20 [2.1e-30]		-0.14 [4.0e-08]	-0.23 [2.7e-31]	-0.07 [5.7e-11]	-0.48 [8.4e-31]	-0.26 [2.7e-25]
kalign	-0.01 [5.8e-02]	0.27 [3.5e-16]	0.34 [2.2e-23]	0.14 [4.0e-08]		-0.09 [6.7e-10]	0.07 [3.4e-02]	-0.34 [2.7e-31]	-0.12 [4.6e-18]
fsa	0.08 [6.8e-19]	0.36 [7.1e-27]	0.43 [2.7e-31]	0.23 [2.7e-31]	0.09 [6.7e-10]		0.16 [1.1e-27]	-0.25 [8.9e-23]	-0.03 [5.5e-04]
clustalo	-0.08 [7.6e-13]	0.20 [7.1e-16]	0.27 [8.9e-31]	0.07 [5.7e-11]	-0.07 [3.4e-02]	-0.16 [1.1e-27]		-0.41 [2.7e-31]	-0.19 [1.3e-28]
tcoffee	0.33 [2.7e-31]	0.61 [2.7e-31]	0.68 [2.7e-31]	0.48 [8.4e-31]	0.34 [2.7e-31]	0.25 [8.9e-23]	0.41 [2.7e-31]		0.22 [4.6e-30]
probcons	0.11 [1.2e-26]	0.39 [1.7e-30]	0.46 [8.1e-31]	0.26 [2.7e-25]	0.12 [4.6e-18]	0.03 [5.5e-04]	0.19 [1.3e-28]	-0.22 [4.6e-30]	
