# Supplementary material for: Accuracy of multiple sequence alignment methods in the reconstruction of transposable element families
Source: NAR Genom Bioinform. 2022 May 17;4(2):lqac040. doi: 10.1093/nargab/lqac040 (PMC9112768; doi:10.1093/nargab/lqac040)
Supplement: lqac040_Supplemental_Files [file lqac040_supplemental_files.zip › Supplemental - S2 - Revision 4.pdf]

S2.1 Parameters for global alignment of consensi with the Needle alignment tool

The scoring scheme used with the Needle program. While the matrix supports the full IUPAC symbols, the consensi produced in this study are only drawn from the alphabet ‘A’,’C’,’G’,’T’ and ‘N’.

Gap Open Penalty: 25
Gap Extension Penalty: 5

Score Matrix:

```
# FREQS A 0.265 C 0.235 G 0.235 T 0.265
    A   R   G   C   Y   T   K   M   S   W   N
A   9   1  -6 -15 -16 -17 -12  -2 -10  -4  -5
R   1   1   1 -15 -15 -16  -6  -6  -6  -7  -5
G  -6   1  10 -15 -15 -15  -2  -10 -2  -10 -5
C -15 -15 -15  10   2  -6  -9  -2  -2  -9  -5
Y -16 -15 -15   1   1   1  -6  -7  -7  -7  -5
T -17 -16 -15  -6   1   9  -2 -12 -11  -4  -5
K -12  -6  -2 -11  -6  -2  -2 -11  -7  -7  -5
M  -2  -6 -10  -2  -7 -12 -11  -2  -7  -7  -5
S  -10 -6  -2  -2  -7 -11  -7  -7  -2  -10 -5
W  -4  -7 -10 -11  -7  -4  -7  -7 -10  -4  -5
N  -5  -5  -5  -5  -5  -5  -5  -5  -5  -5   1
```

S2.2 Evaluation with Charlie1 (DNA transposon) and CR1 (LINE)

Results of the assessment of two additional divergence simulations seeded with the DNA Transposon Charlie1 and the LINE family CR1.

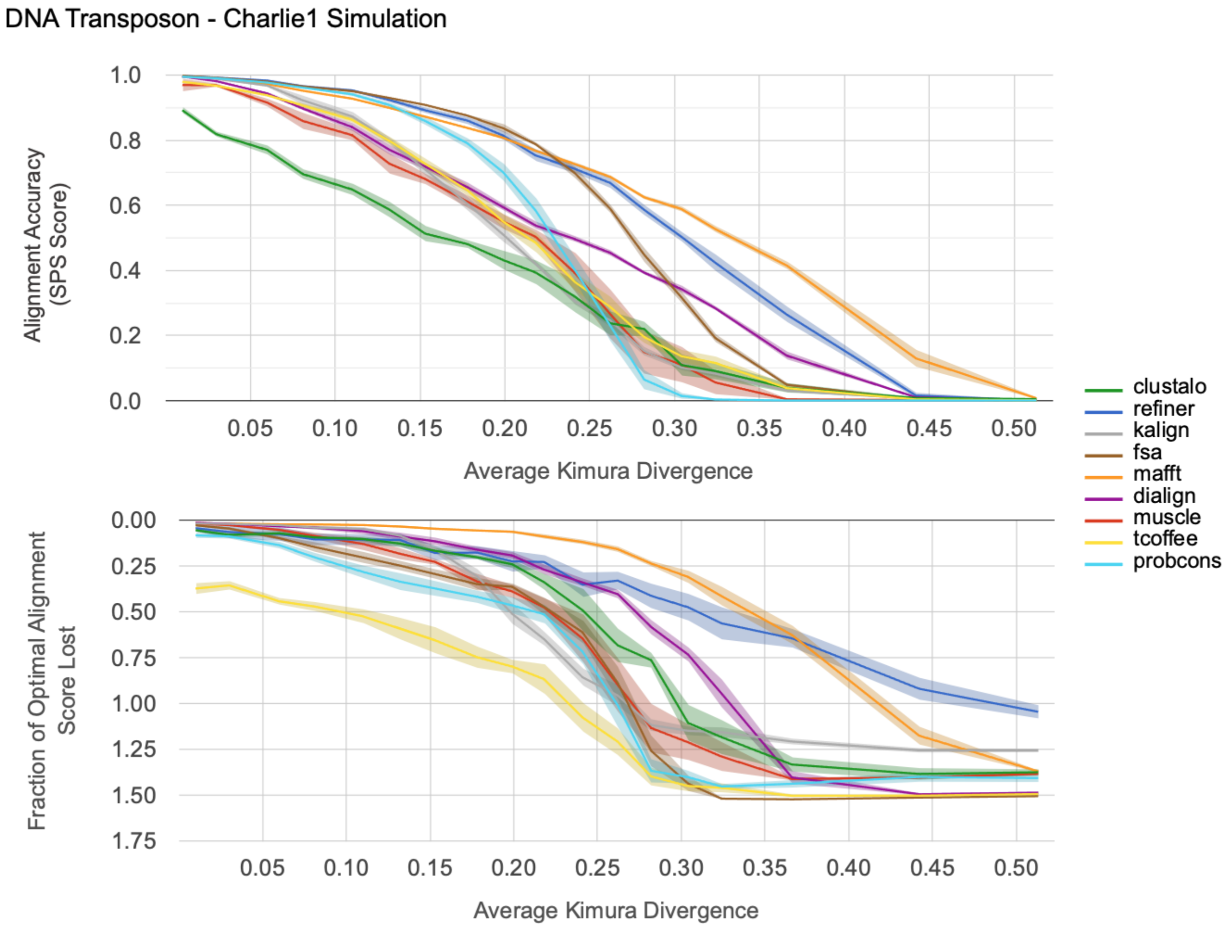

Figure 1.: MSA and derived consensus accuracy with respect to sequence divergence for the alternate DNA transposon family seed Charlie1 (A) The MSA alignment accuracy for each method assessed using the sum-of-pairs (SPS) score over a wide range of sequence divergence. For each tool/divergence combination, 10 replicates were performed. The center line of each band shows the mean SPS score for the tool, while the surrounding shaded region shows the 95% confidence interval. (B) Comparison of the predicted MSA-based consensus with the reference MSA-based consensus,for a range of sequence divergences.. This plot shows the fraction (score(C_r ~ C_r)-score(C_p ~ C_r))/ score(C_r ~ C_r), which corresponds to how effective the computed MSA is at producing a consensus sequence (C_p) that agrees with the one for the simulated sequence (C_r). Scores are for the Needleman-Wunsch (NW) global alignment algorithm (see methods).

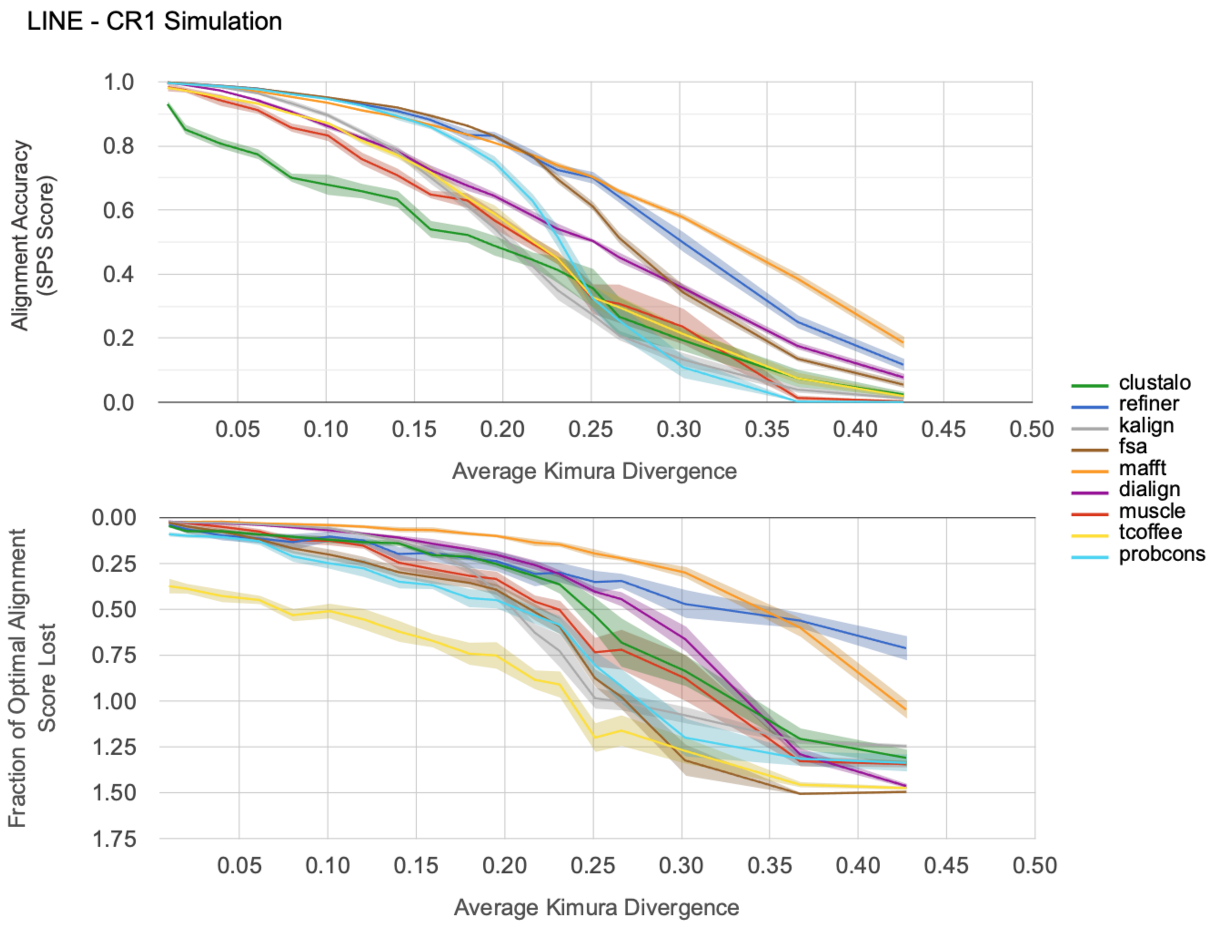

Figure 2.: MSA and derived consensus accuracy with respect to sequence divergence for the alternate LINE family seed CR1 (A) The MSA alignment accuracy for each method assessed using the sum-of-pairs (SPS) score over a wide range of sequence divergence. For each tool/divergence combination, 10 replicates were performed. The center line of each band shows the mean SPS score for the tool, while the surrounding shaded region shows the 95% confidence interval. (B) Comparison of the predicted MSA-based consensus with the reference MSA-based consensus,for a range of sequence divergences.. This plot shows the fraction (score(C_r ~ C_r)-score(C_p ~ C_r))/ score(C_r ~ C_r), which corresponds to how effective the computed MSA is at producing a consensus sequence (C_p) that agrees with the one for the simulated sequence (C_r). Scores are for the Needleman-Wunsch (NW) global alignment algorithm (see methods).

S2.3 Fragmentation Analysis of L2 (LINE)

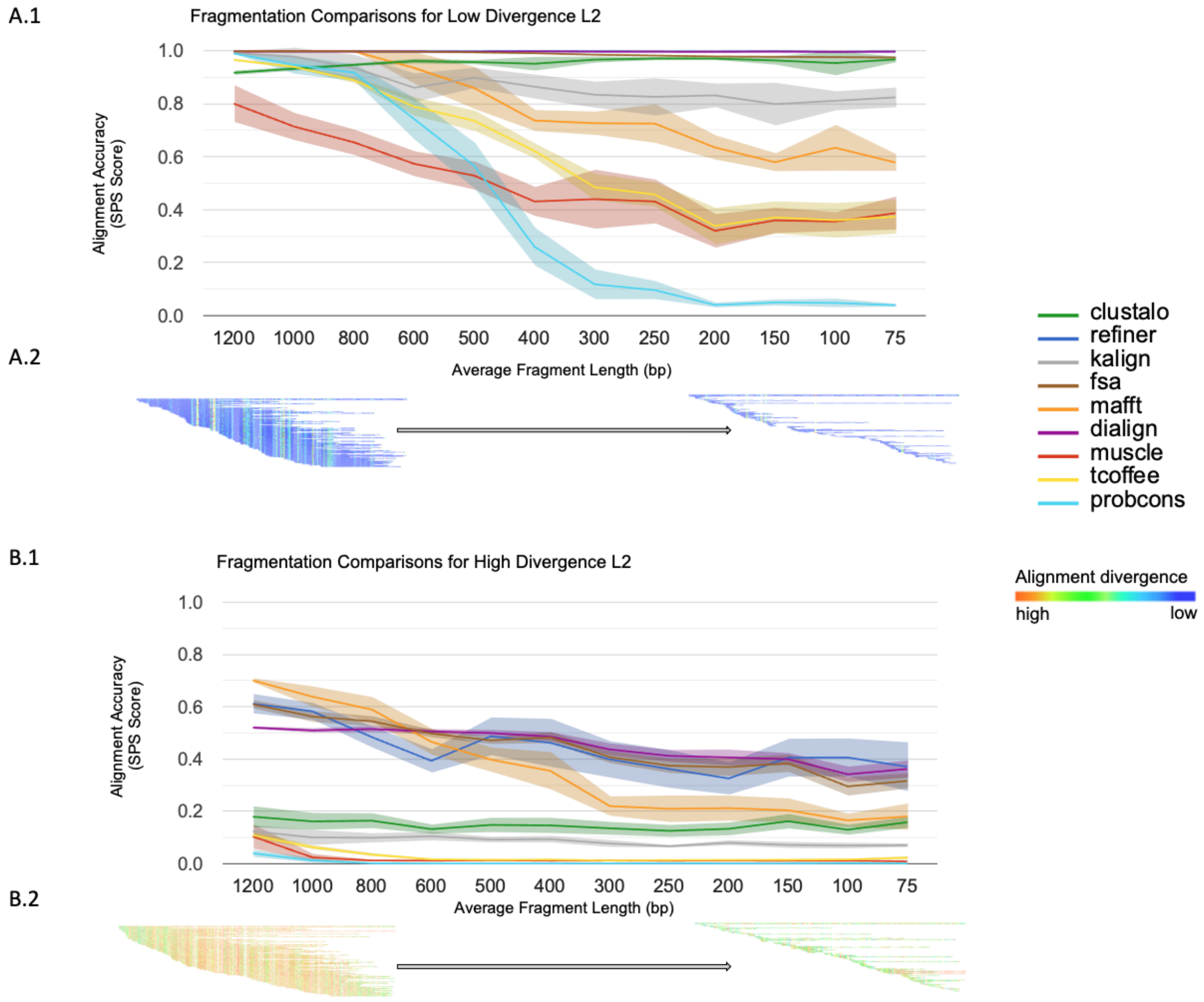

Figure 3: MSA accuracy with respect to sequence fragmentation for simulated L2 LINE sequences. (A.1) The SPS results for simulations of L2 (1% Kimura div) over increasing levels of sequence fragmentation. Fragment lengths are sampled from a distribution around the given mean (x-axis) with a standard deviation of 300. (A.2) A visualization of the fragmentation of the reference MSA for the least fragmented and most fragmented datasets. Each line represents a single fragment; warmer colors represent higher sequence divergence over 10bp windows in the alignment. (B.1) The SPS results for simulations of L2 (28% Kimura div) over increasing levels of sequence fragmentation, with fragment length sampled as above. (B.2) Heatmap visualization of the fragmented MSA, as with (A.2), but for the higher divergence L2 benchmark. Center line of each band shows the mean SPS for each tool, while the surrounding shaded region shows the 95% confidence interval.

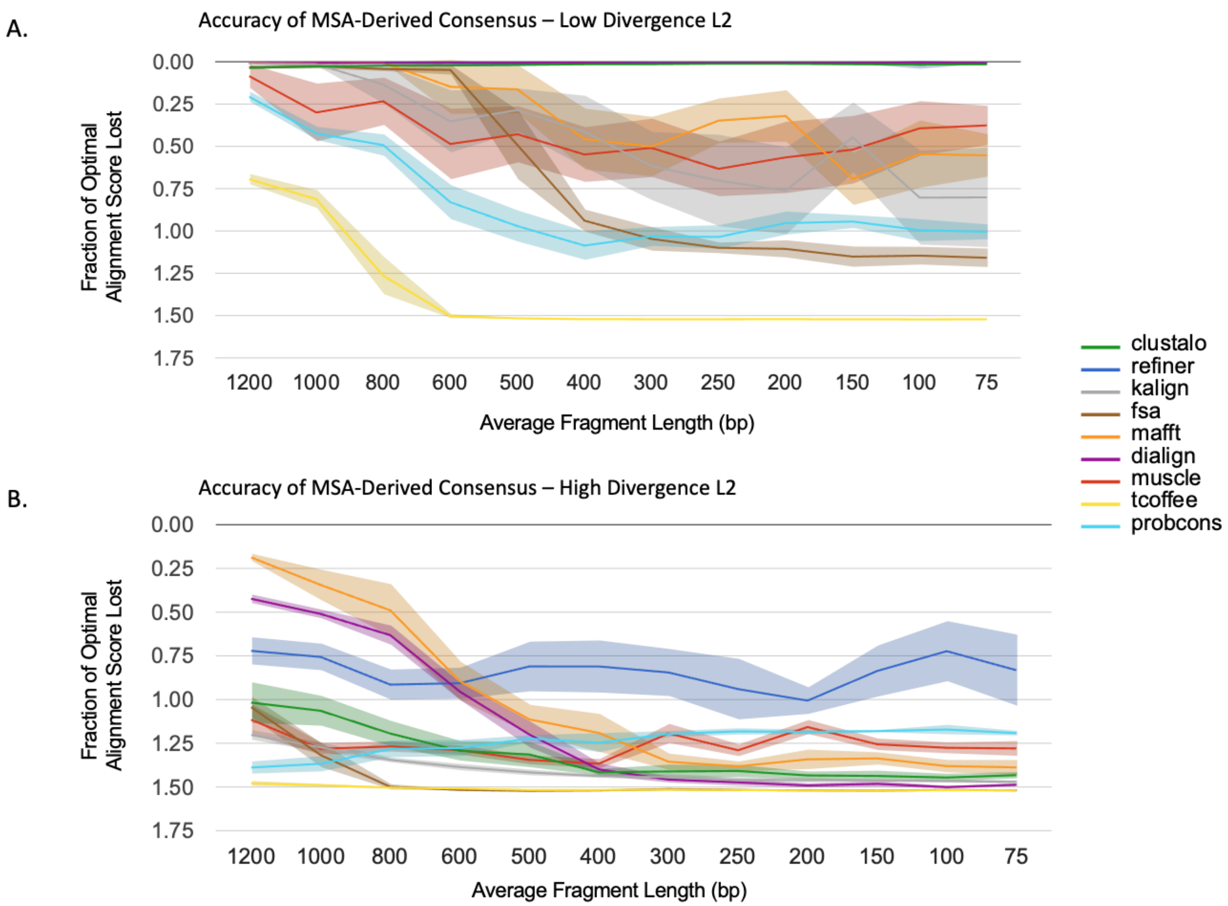

Figure 4: Accuracy of derived consensus with respect to sequence fragmentation for simulated L2 LINE sequences. Comparison of the predicted MSA-based consensus with the reference MSA-based consensus, for fragmented sequences. (A) Fraction of ideal sequence alignment score, as in supplemental Figure 2; input sequences are low-divergence fragments (Avg Kimura div = 1%) as from supplemental Figure 3A. (B) Same as in A, but with high-divergence fragment inputs (Avg Kimura div = 28%) as from supplemental Figure 3B.

S2.4 Short Regions of Misalignment

Short regions of misalignment are not heavily penalized by the sum of pairs (SPS) scoring metric, but they have the potential to have a profound impact on how the MSA is interpreted. For instance, if short misalignments change the interpretation of sequence occupancy for a given column in the alignment, a derived MSA consensus may be significantly shorter. In supplemental figure S1 the simulated MSA is depicted for a highly fragmented but young DNA transposon family. In figure S2 the predicted alignment from the FSA tool is shown for comparison. In this case the misalignment of a majority of the sequences over a 3 bp stretch causes the consensus caller to consider each of these sequences in the occupancy calculation of the subsequent columns. The large gap between these sequences and the remainder of the residues in the alignment cause a large portion of this MSA to be labeled as insertions.

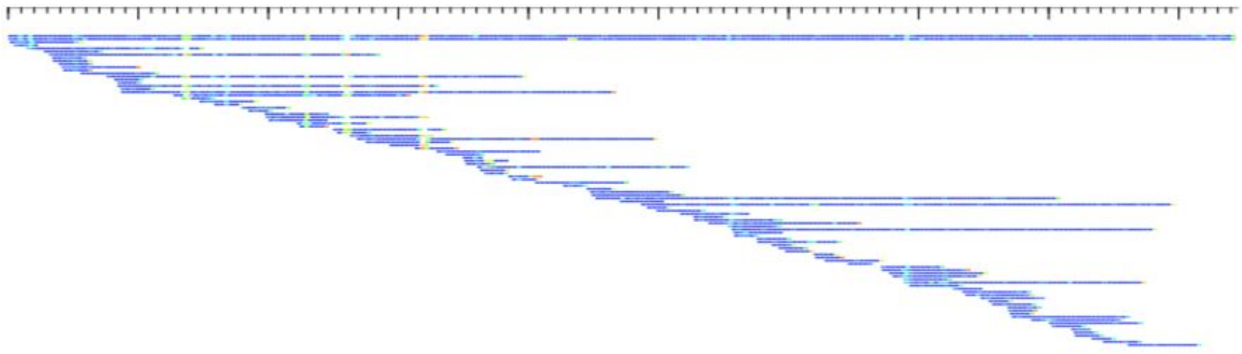

Figure 5: Heatmap depiction of a simulated MSA for a young but highly fragmented DNA Transposon family. Cooler colors represent lower divergence of a sequence relative to the MSA consensus over 10bp non-overlapping windows.

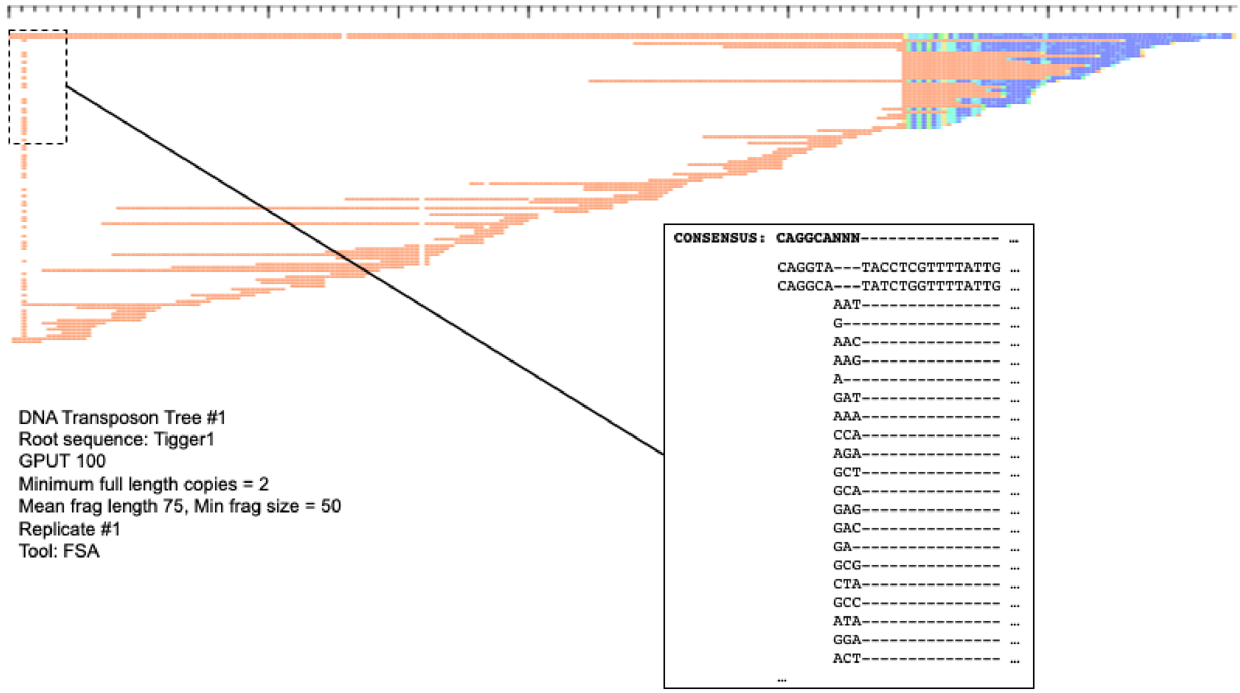

Figure 6: Heatmap depiction of the FSA predicted alignment of the simulated sequences from Figure S1. A portion of the alignment from the upper left corner is shown in detail.

S2.5 Mammalian TE Fragmentation Sizes

The fragment size characteristics of 45 mammalian TE families identified in the human HG38 assembly using the RepeatMasker annotation tool.

Name	Consensus Length	Copies in Genome	Fragments	Longest Fragment (bp)	Mean Fragment Length (bp)	Stdev of Fragment Lengths	Kimura Divergence (ignoring 1 transition at CpG sites)
Arthur1	3947	2135	2840	2236	304.7	232.8	25.5%
Arthur2	3700	805	904	1162	260.8	175	33.0%
BLACKJACK	2969	1361	2037	2689	414	366.9	26.2%
Charlie1	2781	1524	2025	2486	279.9	296.2	21.3%
Charlie24	2449	1211	1388	2056	196.6	225.8	31.3%
Charlie5	2624	2460	3157	2604	208.7	205.1	19.6%
Charlie7	2615	2923	3946	2032	275.6	218.9	28.3%
Charlie8	2457	3214	3820	2153	222.3	161.6	28.9%
Charlie9	2787	1036	1145	2373	189.5	208.1	24.2%
Cheshire	2420	809	1155	2412	351.4	413.7	19.1%
CR1_Mam	2204	1207	1476	1233	217	145.3	36.5%
EuthAT-2	2747	855	1172	2697	328.1	268.1	26.6%
FordPrefect	1683	514	663	1660	496.5	419.4	24.4%
hAT-1_Mam	3469	1098	1399	1797	245.5	179.5	33.9%
hAT-5_Mam	3540	606	707	833	205.6	132.9	32.3%
HSMAR2	1302	1313	1870	1384	609.8	450.2	12.7%
Kanga1	779	680	842	1514	218.8	173.7	25.8%
L1MA1	6302	2865	4641	7805	906.5	1167.3	11.3%
L1MA5	6300	2518	4379	7202	635.7	758.2	16.2%
L1MB1	6168	3428	6482	5677	512	550.9	19.2%
L1MC1	6333	7203	13849	6469	573.5	585.4	17.5%
L1MD1	6242	2662	7427	5904	569.2	575.2	21.0%
L1ME5	6194	2324	3353	5559	337.3	279.9	34.5%
L1PA10	6154	5630	7997	7014	962.5	1179.3	11.5%
L1PA5	6168	9319	11892	6546	1404.3	1793.2	3.8%
L1PB1	6151	7019	13552	7673	962.7	1249.8	9.3%

Name	Consensus Length	Copies in Genome	Fragments	Longest Fragment (bp)	Mean Fragment Length (bp)	Stdev of Fragment Lengths	Kimura Divergence (ignoring 1 transition at CpG sites)
L2-1_AMi	1235	743	773	771	198.5	117.3	38.2%
L2a	3426	76685	154946	3095	270.3	239.8	35.3%
L2b	3375	51557	99144	2884	198.9	162.5	38.7%
L3	4099	26888	41399	1752	199.7	142.4	41.1%
L4_A_Mam	4990	3924	5600	1935	258.9	176.7	36.9%
L4_C_Mam	5138	2979	3976	1553	222	155.3	37.5%
Looper	1557	356	591	1559	331.3	298.6	21.2%
MamRTE1	3812	4590	5427	1447	164.7	117.4	39.2%
OldhAT1	2842	2090	2169	861	95.8	76.8	34.5%
Penelope1_Vert	1079	700	725	547	89.6	69.3	31.3%
Tigger1	2418	7826	13290	2490	718.8	672.3	13.0%
Tigger10	2097	1034	1234	1296	221.9	159.9	35.5%
Tigger2	2718	2516	3432	2795	490.8	589.2	12.6%
Tigger3	3029	1057	1409	3027	353.4	393.7	13.0%
Tigger7	2492	2983	3354	2464	221.8	198.5	13.7%
Tigger8	667	742	883	650	260.2	148.9	30.0%
Zaphod	4079	1618	2267	3649	318.1	271.4	24.3%
Zaphod2	3592	602	778	1786	223.4	221.8	24.4%
Zaphod3	2624	909	1098	1552	209.8	178.4	22.5%

S2.6 Column Score Metric

The Column Score (CS) metric is often provided as a specificity measure for MSA prediction. The column score is the fraction of aligned columns that were correctly reproduced in the predicted alignment. A single incorrectly aligned base will invalidate a column, therefore the expectation is the CS score will drop precipitously with highly diverged sequences. The CS results for the four simulated families are plotted below.

(A) Tigger1 Simulation

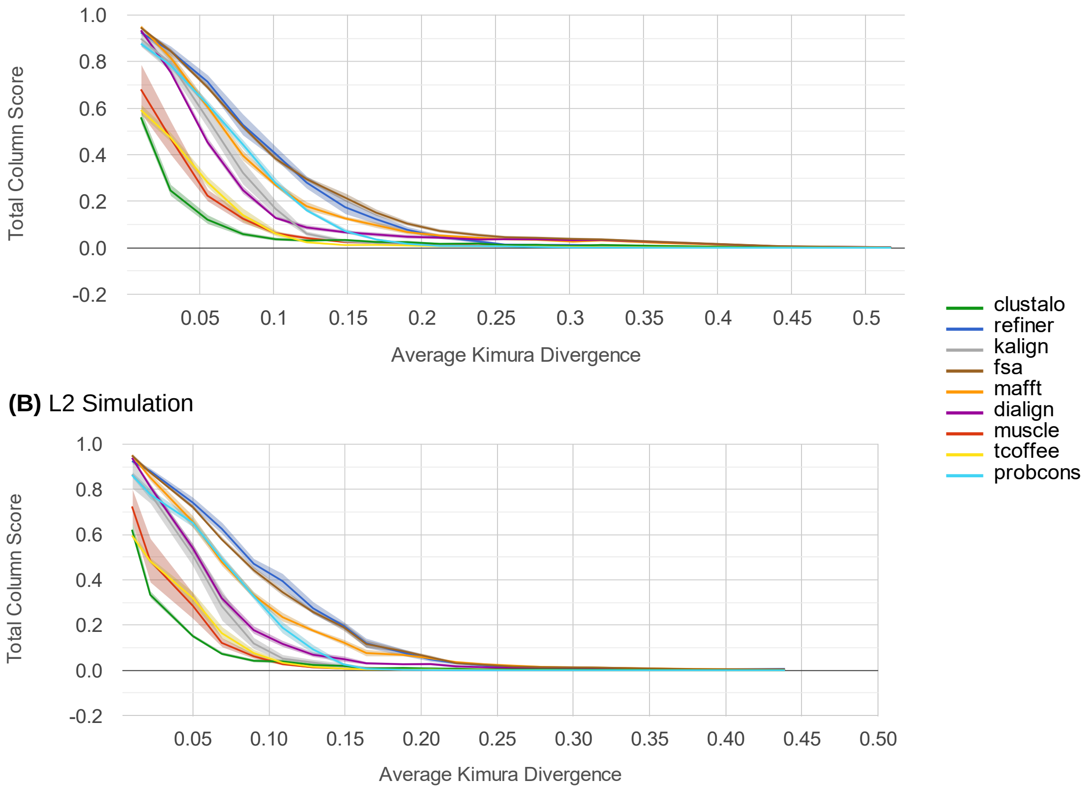

Figure 7: Column Score with respect to sequence divergence for the Tigger1 and L2 families. The MSA alignment column score (CS) for each method assessed over a wide range of sequence divergence. For each tool/divergence combination, 10 replicates were performed. The center line of each band shows the mean SPS score for the tool, while the surrounding shaded region shows the 95% confidence interval.

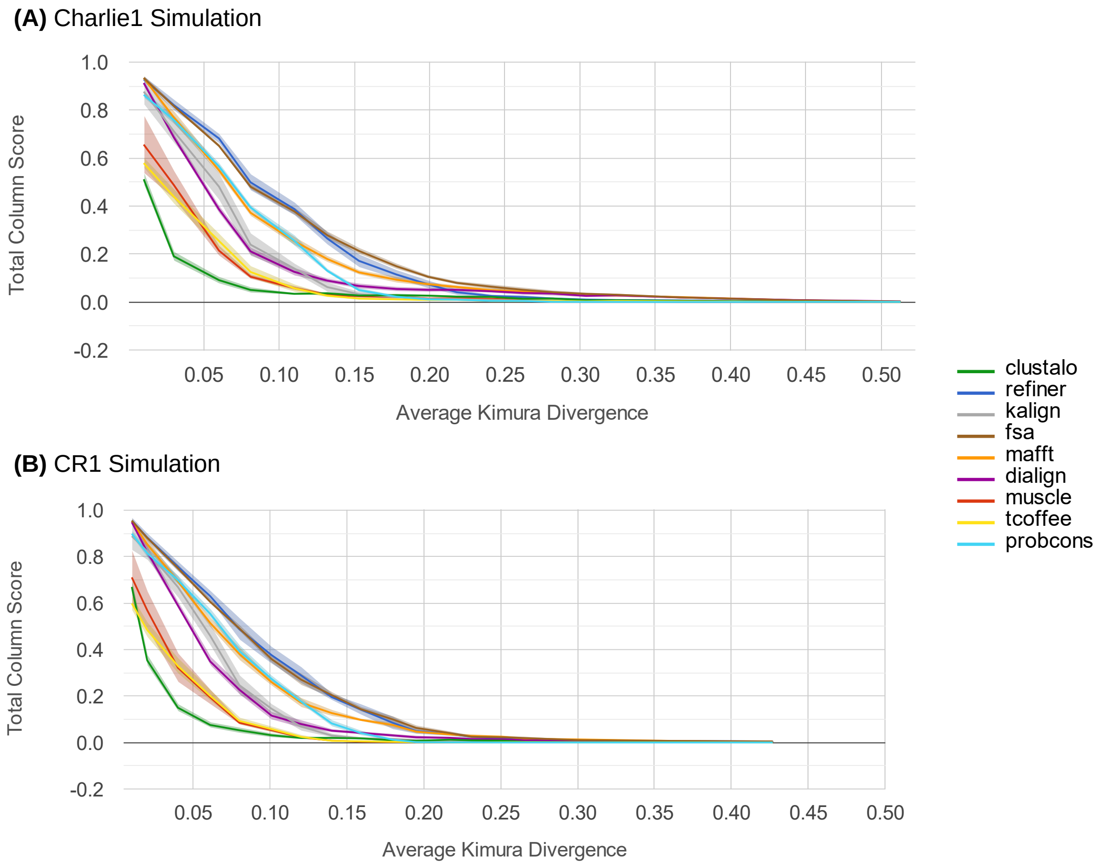

Figure 8: Column Score with respect to sequence divergence for the Charlie1 and CR1 families. The MSA alignment column score (CS) for each method assessed over a wide range of sequence divergence. For each tool/divergence combination, 10 replicates were performed. The center line of each band shows the mean SPS score for the tool, while the surrounding shaded region shows the 95% confidence interval.

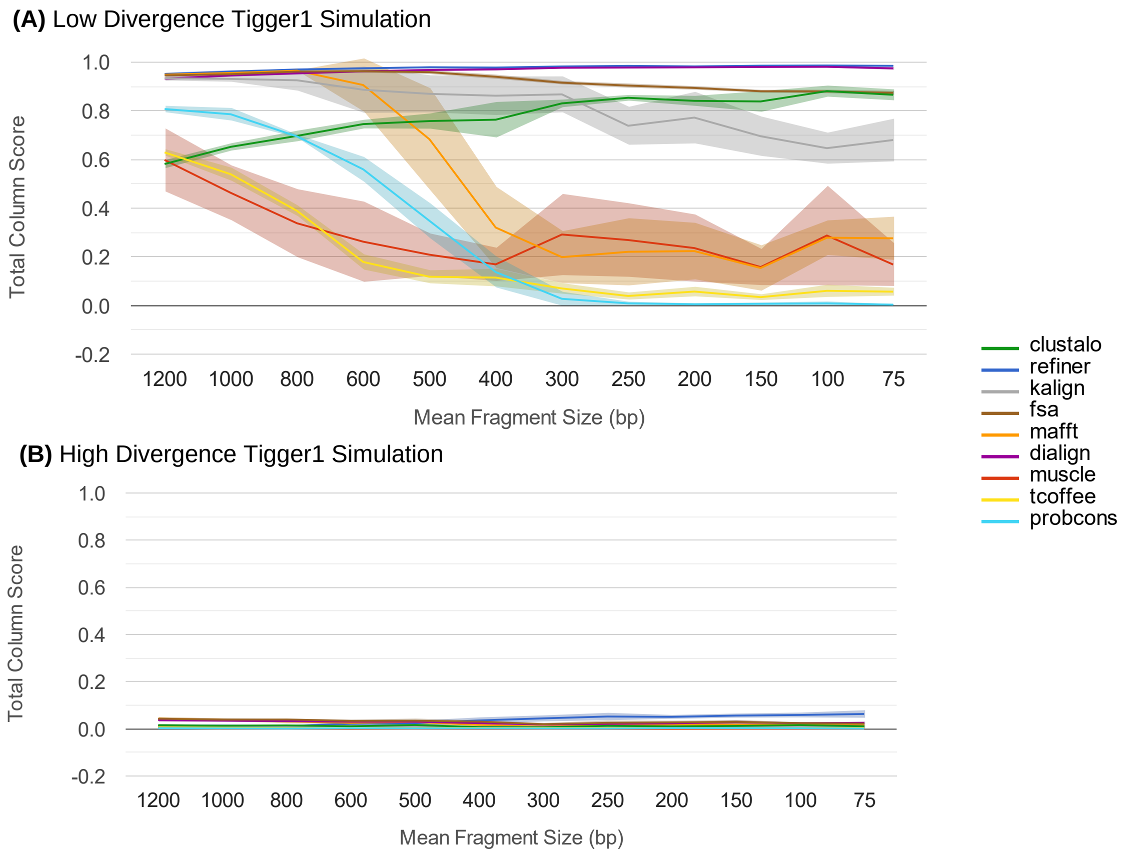

Figure 9: Column Score with respect to sequence fragmentation for low and high divergence Tigger1 simulations. (A) The MSA alignment column score (CS) for each method assessed over increasing levels of sequence fragmentation in Tigger1 simulations (1% Kimura divergence). Fragment lengths are sampled from a distribution around the given mean (x-axis) with a standard deviation of 300. For each tool/divergence combination, 10 replicates were performed. The center line of each band shows the mean SPS score for the tool, while the surrounding shaded region shows the 95% confidence interval. (B) The CS results for simulations of Tigger1 (28% Kimura divergence) over increasing levels of sequence fragmentation, with fragment length sampled as above.

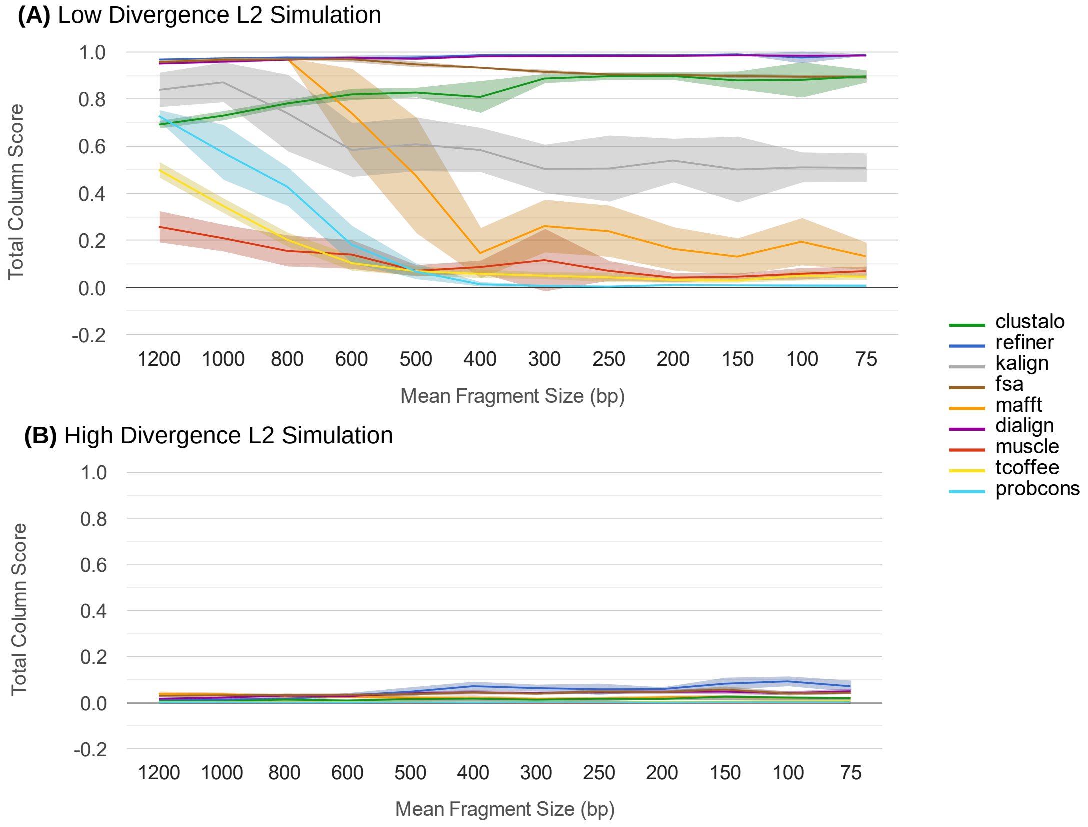

Figure 10: Column Score with respect to sequence fragmentation for low and high divergence L2 simulations. (A) The MSA alignment column score (CS) for each method assessed over increasing levels of sequence fragmentation in L2 simulations (1% Kimura divergence). Fragment lengths are sampled from a distribution around the given mean (x-axis) with a standard deviation of 300. For each tool/divergence combination, 10 replicates were performed. The center line of each band shows the mean SPS score for the tool, while the surrounding shaded region shows the 95% confidence interval. (B) The CS results for simulations of L2 (28% Kimura divergence) over increasing levels of sequence fragmentation, with fragment length sampled as above.
